# Associations of health parameters at 16 years of age with structural knee MRI findings at 33 years of age in a general population-based birth cohort

**DOI:** 10.1016/j.ocarto.2026.100780

**Published:** 2026-03-14

**Authors:** Antti Kemppainen, Joona Tapio, Miika T. Nieminen, Simo Saarakkala, Mika T. Nevalainen

**Affiliations:** a Research Unit of Health Sciences and Technology, Faculty of Medicine, University of Oulu, P.O. Box 5000, FI-90014 Oulu, Finland; b Department of Diagnostic Radiology, Oulu University Hospital, P.O. Box 50, FI-90029 Oulu, Finland; c Faculty of Biochemistry and Molecular Medicine, University of Oulu, P.O. Box 5400, FIN-90014 Oulu, Finland; d Biocenter Oulu, University of Oulu, P.O. Box 5400, FIN-90014 Oulu, Finland; e Medical Research Center Oulu, University of Oulu and Oulu University Hospital, Oulu, Finland

**Keywords:** Adolescent, Cohort studies, Knee joint /diagnostic imaging, Longitudinal studies, Magnetic resonance imaging, Osteoarthritis

## Abstract

**Objective:**

To evaluate the potential associations of clinical health parameters at 16 years and knee magnetic resonance imaging (MRI) findings at 33 years in a random subpopulation of the Northern Finland Birth Cohort 1986.

**Method:**

Subjects with knee MRI data at 33 years (*n* = 288) and available clinical and questionnaire data at both 16 years (minimum *n* = 236) and 33 years (*n* = 288) were included. The MRI Osteoarthritis Knee Score (MOAKS) system was used for MRI grading. The associations of clinical and background parameters at the 16-year time point, and their change from 16- to 33-year time point, were evaluated in relation to individual MRI findings.

**Results:**

Higher body mass, waist and hip circumference at 16 years and increased body mass between time points were associated with MRI findings such as bone marrow lesions, cartilage lesions and osteophytes. An increase in systolic blood pressure (ΔSBP) and fasting glucose (ΔfP-glucose) between time points were associated with MRI findings, mediated through higher body mass index (BMI). The associations of ΔfP-glucose were limited to osteophytes and mediated but not dependent on BMI.

**Conclusion:**

Higher body mass, waist and hip circumference at 16 years were most frequently associated with several imaging findings and mediated most other associations. Moreover, impaired glucose metabolism could be independently linked to increased osteophyte formation. Although causality is not demonstrated, higher body mass and circumferential measurements with their related co-morbidities seem to significantly contribute to pathogenesis of osteoarthritis already from adolescence.

## 1 Introduction

Osteoarthritis (OA) is a leading cause of pain and functional impairment in working age adults and the elderly [1,2] with substantial observed and projected increase in the global incidence [3,4]. Previously thought to mainly affect older populations, the prevalence of OA has also risen in young adults [1,3,4] with the knee joint being most often affected [1]. In addition to old age, established risk factors for knee OA include metabolic syndrome [5,6], obesity [1,7,8], family history [1,8], physically demanding work [7,9], knee injuries and athletic background [7,10,11].

Amongst preventable risk factors, obesity is the most important [1,7,8]. According to World Health Organization (WHO), in 2022 43% of adults globally were overweight [12]. Even more alarmingly, 2017–2018 National Health and Nutrition Examination Survey data showed 25.4% of 2-19-years-olds in the US being either obese or severely obese, and 16.1% being overweight [13]. Although childhood obesity has been shown to predict adult obesity [[14], [15], [16]], only few studies have been published on the effects of childhood obesity in developing knee OA in young adulthood. Childhood obesity has been previously associated with knee pain, stiffness, and dysfunction scores [17], patellofemoral cartilage lesions and bone marrow lesions (BMLs) and tibiofemoral BMLs in males but not tibiofemoral cartilage lesions in adulthood [18]. Patellofemoral and medial femoral regions would be the expected sites for chondral lesions in knee OA [[19], [20], [21]]. Besides body weight, the significance of other health metrics in childhood have not been extensively studied [22].

Northern Finland Birth Cohorts (NFBCs) are large ongoing population cohort studies with regular follow-ups since the antenatal period. In NFBC1986 33-year follow-up, unilateral knee joints of a random subpopulation of relatively healthy participants were imaged with MRI. Amongst other imaging findings, small cartilage lesions and osteophytes were common, and few participants already exhibited definite [23] knee OA. Higher BMI was most frequently associated with MRI findings [8]. The previous data collection prior to the 33-year collection was at the age of 15–16 years, when questionnaire and clinical data, including physical health and anthropometric measurements, were collected.

Previous research on childhood health and the development of knee OA in adulthood is limited and has mostly focused on body weight. The purpose of this study was to evaluate the potential associations of identifiable health traits in adolescence with structural MRI findings and developing knee OA in young adulthood, using the available NFBC1986 data.

## 2 Method

### 2.1 Study population

NFBC1986 is a longitudinal general population-based birth cohort including 99% of children born in the two northernmost provinces of Finland between July 1985 and June 1986 (*n* = 9432) [24]. At 16 years of age, questionnaires were sent to participants with known addresses (*n* = 9215, 97.2%), and data was received from 7182 participants (77.9%). Clinical data was collected from 6795 participants (73.7%). The project is ongoing, with the latest collection at ages 33–35, involving clinical examinations (*n* = 1807) and postal questionnaires (*n* = 2347) [25].

A flow-chart of the study setup is presented in Fig. S1. Inclusion criteria were available knee MRI data at 33 years of age (*n* = 288, 38.9% males) and available clinical and questionnaire data at both 16 years of age (minimum *n* = 236) and at 33 years of age (*n* = 288).

The NFBC1986 study was conducted according to the declaration of Helsinki and approved by the ethical committee of Northern Ostrobothnia Hospital District in Oulu, Finland. Participants provided written informed consent. The authors obtained permission for this study from the NFBC (project number P1215). The study adheres to the STROBE guidelines.

### 2.2 Background information

Subjects self-reported their smoking status, physical activity and alcohol consumption at age 16. Physical activity score was based on 4 questions about the duration and frequency of functional (1–5, 1–6) and leisure time exercise (1–8, 1–6). The sum of the 4 questions was used in the analyses. Smoking status was categorized as “Never” or “Ever” based on a questionnaire. Alcohol consumption was classed as “Abstinent” (never or tried once), “Occasional” (once a month at most) or “regular” (multiple times a month or more). Background parameters at age 33 were determined as described in Ref. [8].

### 2.3 Clinical examinations and laboratory analyses

At both time points, participants were informed to abstain from caffeine and smoking before the clinical examinations. Trained nurses measured body weight and height, waist and hip circumferences as well as brachial systolic and diastolic blood pressure (SBP and DBP, respectively) and heart rate (HR). Body mass index (BMI) was calculated.

Laboratory analyses were conducted after an overnight fast. At age 16, laboratory measures were analyzed using Cobas Integra 700 automatic analyzer (Roche Diagnostics, Basel, Switzerland) at Oulu University Hospital laboratory within 24 h of sampling [26]. The determination of laboratory parameters at age 33 has been recently described [8].

### 2.4 Magnetic resonance imaging

Knee MRI examinations and their semi-quantitative assessment were performed according to previously detailed methods. Briefly, a single knee joint was scanned for every participant using a 3T MRI scanner with uniform imaging protocols: turbo spin-echo T2 sequence with fat saturation, turbo spin-echo proton density weighted sequence and double echo steady state sequence [8]. Images were evaluated using the MRI Osteoarthritis Knee Score (MOAKS) system [27,28]. The scoring was done by a board-certified radiologist and musculoskeletal radiology fellow (AK) with five years of training, following calibration sessions with a board-certified, fellowship-trained musculoskeletal radiologist (MTNe) who had a decade of experience.

### 2.5 Statistical methods

Subjects with missing data were omitted from individual analyses. Continuous variables were checked for skewness. Normally distributed continuous variables are presented as mean (M) and standard deviation, and non-normally distributed continuous variables are presented as median (Mn) and interquartile range. Count data is presented as number of observations (n) and percentage.

Clinical and background data were first evaluated in descriptive statistics at both time points separately for the whole population, males and females. The change of these parameters from 16 years of age to 33 (Δparameter) were calculated for parameters with comparable data available at both time points.

Each parameter at 16 years of age, and the Δparameter, were then individually evaluated in crude unadjusted regression models with each individual knee MRI finding. In case of a binary outcome, a logistic regression model was used, and a Poisson regression model with robust standard errors was used for multi-classed outcomes. For colinear variables (such as SBP and DBP), only one of the parameters is reported.

Based on unadjusted regression analyses and descriptive data, risk estimates of selected clinical parameters at age 16 on the knee MRI findings, were evaluated in three further multivariable models. Model 1 included sex and BMI (at age 16) as explanatory factors and was conducted without additional covariates and for ΔBMI, ΔfP-glucose and ΔSBP. The aim of Model 1 was to evaluate whether the detected associations were influenced by BMI at age 16 and sex. Model 2 included sex and BMI (at age 33) as explanatory factors and was conducted for BMI (at age 16). The aim of the model was to evaluate how the association of BMI at age 16 was influenced by BMI at age 33. Model 3 included sex and BMI (at age 16) and the given parameter at age 16 as explanatory variables and was conducted for ΔfP-glucose and ΔSBP. The aim of the model was to evaluate whether the associations of the Δparameters were influenced by the baseline value of the given Δparameter.

To validate results from the regression analyses, we evaluated clinical data at both time points and the change in these parameters from age 16 to 33 was evaluated in BMI classes [12] and according to ΔfP-glucose solely for descriptive purposes. These classes were not used in regression analyses.

Statistical analyses were performed using IBM SPSS statistics, version 29.0.0.0 (IBM Corp, Armonk, NY).

## 3 Results

### 3.1 Background and clinical characteristics of the study population at 16 and 33 years of age

Background and clinical characteristics of the study population at 16 and 33 years of age are presented in Table 1. Most background characteristics were not available for analysis at both time points (Table 1). 61.1 % of the participants were female (*n* = 176). At 33 years of age, most participants had none-to-mild clinical knee symptoms of knee pain, stiffness, or function. Depending on the parameter, most parameters at both time points were available for a total of 235–262 participants (Table 1).

**Table 1:** Background and clinical characteristics of the study population at 16 and 33 years of age and change in the characteristics between the two timepoints.

	At age 16		At age 33		Delta parameter	
	n	M (SD)/Mn(IQR)	n	M (SD)/Mn(IQR)	n	M (SD)/Mn(IQR)
		or n (%)		or n (%)		or n (%)
**Background characteristics**						
Participants	288	288 (100.0)	288	288 (100.0)	N/A	N/A
Males n (%)	288	112 (38.9)	288	112 (38.9)	N/A	N/A
Females n (%)	288	176 (61.1)	288	176 (61.1)	N/A	N/A
Age (years)	256	16.0 (0.3)	288	33.7 (0.4)	N/A	N/A
Prior lower limb fracture n (%)	NA	NA	287	37 (12.8)	NA	NA
Family history of knee OA n (%)	NA	NA	288	93 (32.3)	NA	NA
Ever smoker n (%)	262	154 (53.5)	288	178 (61.8)	262	24 (8.3)
Alcohol: Abstinent n (%)	265	152 (52.8)	NA	NA	NA	NA
Alcohol: Occasional n (%)	265	92 (31.9)	NA	NA	NA	NA
Alcohol: Regular n (%)	265	21 (7.3)	NA	NA	NA	NA
Alcohol: g/week	NA	NA	254	16.5 (10.5–34.9)	NA	NA
Physical activity score	265	15.4 (4.1)	288	14.9 (3.3)	NA	NA
WOMAC pain	NA	NA	284	2.6 (4.2)	NA	NA
WOMAC stiffness	NA	NA	284	1.1 (2.1)	NA	NA
WOMAC function	NA	NA	284	5.0 (10.7)	NA	NA
WOMAC total	NA	NA	284	8.5 (15.8)	NA	NA
**Clinical characteristics**						
BMI (kg/m^2^)	257	21.1 (3.6)	288	25.7 (4.6)	257	4.6 (3.7)
Height (cm)	257	169.2 (8.7)	288	171.2 (9.4)	257	2.4 (4.1)
Weight (kg)	260	60.5 (11.9)	288	75.7 (15.8)	260	15.6 (10.7)
Waist circumference (cm)	256	73.1 (8.5)	287	87.0 (13.1)	255	14.1 (11.1)
Hip circumference (cm)	257	92.9 (7.2)	287	99.7 (11.5)	256	6.8 (9.8)
Systolic blood pressure (mmHg)	255	116.5 (11.5)	288	112 (12.2)	255	−4.5 (17.3)
Diastolic blood pressure (mmHg)	255	68.9 (7.2)	288	74.2 (8.9)	255	5.3 (11.9)
Heart rate (bpm)	255	68.2 (10.0)	288	72 (12)	255	3.6 (16.0)
fP-glucose (mmol/L)	236	5.2 (0.9)	287	5.0 (0.6)	235	−0.2 (0.5)
fP-total cholesterol (mmol/L)	240	4.3 (0.8)	288	4.7 (0.9)	240	0.3 (0.9)
fP-HDL cholesterol (mmol/L)	240	1.4 (0.3)	288	1.5 (0.3)	240	0.1 (0.3)
fP-LDL cholesterol (mmol/L)	240	2.3 (0.6)	288	2.7 (0.8)	240	0.4 (0.7)
fp-triglycerides	240	0.8 (0.4)	288	1.0 (0.7)	240	0.1 (0.7)
P-Urate (umol/L)	NA	NA	288	304.4 (70.3)	NA	NA
CRP (mg/L)	245	0.7 (1.3)	288	0.8 (0.4–1.6)	245	0.9 (3.0)

Data is presented as mean (M) and standard deviation (SD) for normally distributed continuous variables, median (Mn) and interquartile range (IQR) for skewed continuous variables and count (n) and percentage (%) for count variables. M; Mean, SD; Standard Deviation, OA; osteoarthritis, BMI; Body Mass Index, fP-; fasted plasma, HDL; High-Density Lipoprotein, LDL; Low-Density Lipoprotein, hs-CRP: high-sensitivity C-Reactive Protein.

From age 16 to 33, the mean increase in BMI was +4.6 kg/m^2^, with similar increases observed for both sexes. Accordingly, both mean weight (+15.6 kg) and waist cx. (+14.1 cm) were also increased with larger magnitudes observed in males compared to females. Smaller increases were observed in height (+2.4 cm), and hip cx (+6.8 cm) but similarly these were more prominently observed in male participants (Tables 1, S1-S2).

For males, both SBP (+4.6 mmHg) and DBP (+7.4 mmHg) increased but in females SBP decreased (−10.7 mmHg) and DBP increased (+3.8 mmHg) from 16 to 33 years of age. An increase in HR (+5.5 bpm) was only observed in females (Tables S1–S2).

Of laboratory measures, the greatest increases were observed in LDL and total cholesterol in males, with smaller changes observed for fasting glucose, triglycerides, HDL cholesterol and CRP levels (Tables 1, S1-S2).

### 3.2 Structural knee MRI findings of the study population at 33 years of age

Structural knee MRI findings of the study population at 33 years of age are presented in Tables S3–S4. These have been previously published and discussed in detail [8]. In short, detected articular cartilage lesions were mostly small and identified in 56.2% (*n* = 162) of patellofemoral and 25.3% (*n* = 63) of tibiofemoral joints. Full-thickness cartilage lesions and bone marrow lesions (BMLs) were sparse and mostly located in the patellofemoral joint. Osteophytes, mostly small or doubtful, were detected in 51.7% (*n* = 146) of patellofemoral and 17.4% (*n* = 41) of tibiofemoral joints. Meniscal and cruciate ligament pathologies were rare (Tables S3–S4).

### 3.3 Unadjusted associations of clinical parameters at 16 years of age with structural knee MRI findings at 33 years of age

Unadjusted associations of clinical parameters at 16 years of age with structural knee MRI findings at 33 years of age are presented in Tables 2 and S5. BMI, weight and waist and hip cx. at 16 years of age were associated with articular cartilage loss, most full thickness cartilage losses, most BMLs and osteophytes at 33 years of age, whereas height was not as prominently associated with most knee MRI findings (Table 2). For other MRI parameters of interest, most anthropometrics were associated with ganglion cysts and knee joint effusion (Table 2).

**Table 2:** Unadjusted Relative Risks or Odds Ratios with 95% Confidence Intervals of individual clinical parameters at age 16 for knee MRI findings at age 33.

	Male sex	BMI(kg/m^2^)	Height (cm)	Weight (kg)	Hip cx. (cm)	Waist cx. (cm)
**Cartilage loss**						
Tibial medial	0.79 (0.26–2.40)	1.11 (1.05–1.18)	1.04 (1.00–1.09)	1.04 (1.02–1.06)	1.08 (1.04–1.13)	1.06 (1.02–1.09)
Tibial lateral	1.68 (0.79–3.57)	1.10 (1.04–1.16)	1.07 (1.02–1.11)	1.04 (1.03–1.05)	1.07 (1.04–1.10)	1.06 (1.04–1.08)
Femoral medial	1.11 (0.65–1.88)	1.08 (1.03–1.13)	1.02 (0.99–1.05)	1.03 (1.02–1.04)	1.05 (1.03–1.07)	1.04 (1.02–1.06)
Femoral lateral	2.44 (0.89–6.75)	1.08 (1.01–1.15)	1.06 (1.02–1.12)	1.03 (1.01–1.06)	1.05 (1.01–1.10)	1.05 (1.02–1.08)
Tibiofemoral	1.26 (0.81–1.95)	1.07 (1.03–1.11)	1.02 (1.00–1.05)	1.03 (1.01–1.04)	1.05 (1.03–1.07)	1.04 (1.02–1.05)
Patellofemoral	0.94 (0.73–1.21)	1.03 (1.00–1.05)	1.00 (0.99–1.02)	1.01 (1.00–1.02)	1.01 (0.99–1.03)	1.01 (1.00–1.02)
Any	1.04 (0.83–1.29)	1.03 (1.00–1.05)	1.01 (0.99–1.02)	1.01 (1.00–1.02)	1.01 (0.99–1.02)	1.01 (1.00–1.02)
**FT cartilage loss**						
Tibial lateral∗	0.31 (0.06–1.72)	1.03 (0.84–1.28)	1.10 (0.99–1.22)	1.03 (0.98–1.09)	1.04 (0.94–1.15)	1.04 (0.96–1.12)
Femoral medial	1.05 (0.26–4.18)	1.10 (1.01–1.21)	1.01 (0.92–1.11)	1.03 (1.00–1.07)	1.07 (0.99–1.15)	1.06 (1.01–1.10)
Femoral lateral	2.36 (0.49–11.28)	1.08 (0.99–1.18)	1.11 (1.04–1.18)	1.04 (1.02–1.06)	1.07 (1.00–1.14)	1.04 (1.00–1.08)
Tibiofemoral	1.57 (0.59–4.15)	1.08 (1.00–1.16)	1.05 (0.98–1.12)	1.03 (1.01–1.06)	1.06 (1.01–1.11)	1.05 (1.02–1.08)
Patellofemoral	1.18 (0.59–2.34)	1.10 (1.05–1.16)	1.02 (0.99–1.06)	1.04 (1.03–1.04)	1.06 (1.03–1.09)	1.05 (1.04–1.07)
Any	1.38 (0.78–2.45)	1.08 (1.03–1.14)	1.03 (0.99–1.07)	1.03 (1.02–1.04)	1.05 (1.02–1.08)	1.05 (1.03–1.06)
**Size of BML**						
Tibial lateral∗	0.12 (0.01–1.06)	1.06 (0.89–1.26)	1.14 (1.03–1.27)	1.04 (1.00–1.09)	1.05 (0.97–1.15)	1.05 (0.98–1.12)
Femoral medial	1.05 (0.20–5.36)	1.13 (1.04–1.22)	1.03 (0.95–1.11)	1.04 (1.01–1.07)	1.08 (1.02–1.15)	1.07 (1.03–1.11)
Femoral lateral	3.14 (0.51–19.36)	1.06 (0.96–1.17)	1.14 (1.04–1.26)	1.04 (1.02–1.07)	1.04 (0.96–1.13)	1.04 (1.00–1.08)
Tibiofemoral	1.73 (0.60–4.96)	1.10 (1.03–1.18)	1.08 (1.02–1.15)	1.04 (1.02–1.06)	1.07 (1.03–1.12)	1.06 (1.03–1.10)
Patellofemoral	1.39 (0.62–3.10)	1.07 (1.01–1.14)	1.01 (0.97–1.04)	1.02 (1.01–1.04)	1.03 (0.98–1.08)	1.04 (1.01–1.06)
Any	1.70 (0.91–3.20)	1.07 (1.02–1.13)	1.03 (1.00–1.07)	1.03 (1.02–1.04)	1.04 (1.01–1.08)	1.04 (1.02–1.06)
**% that is BML**						
Tibial lateral∗	0.12 (0.01–1.06)	1.06 (0.89–1.26)	1.14 (1.03–1.27)	1.04 (1.00–1.09)	1.05 (0.97–1.15)	1.05 (0.98–1.12)
Femoral medial	1.29 (0.29–5.68)	1.12 (1.02–1.22)	1.03 (0.95–1.13)	1.04 (1.01–1.07)	1.09 (1.01–1.17)	1.06 (1.02–1.10)
Femoral lateral	2.36 (0.40–13.89)	1.08 (1.01–1.16)	1.13 (1.02–1.26)	1.04 (1.02–1.07)	1.06 (1.00–1.12)	1.05 (1.01–1.09)
Tibiofemoral	2.12 (0.81–5.56)	1.09 (1.02–1.16)	1.10 (1.03–1.16)	1.04 (1.02–1.07)	1.07 (1.03–1.12)	1.06 (1.03–1.09)
Patellofemoral	1.03 (0.46–2.31)	1.09 (1.04–1.15)	1.01 (0.97–1.05)	1.03 (1.02–1.04)	1.06 (1.02–1.09)	1.05 (1.03–1.07)
Any	1.52 (0.83–2.76)	1.08 (1.04–1.13)	1.04 (1.01–1.08)	1.03 (1.02–1.05)	1.05 (1.03–1.08)	1.05 (1.03–1.06)
**Osteophytes**						
Tibial medial	1.15 (0.42–3.14)	1.12 (1.05–1.18)	1.03 (0.96–1.11)	1.04 (1.02–1.06)	1.08 (1.03–1.12)	1.05 (1.02–1.08)
Tibial lateral	1.18 (0.48–2.89)	1.14 (1.09–1.19)	1.02 (0.95–1.10)	1.04 (1.02–1.07)	1.09 (1.05–1.13)	1.06 (1.03–1.09)
Femoral medial	1.45 (0.53–3.96)	1.11 (1.04–1.17)	1.05 (0.99–1.12)	1.04 (1.02–1.06)	1.07 (1.02–1.12)	1.05 (1.02–1.08)
Femoral lateral	1.01 (0.52–1.95)	1.10 (1.06–1.15)	1.01 (0.96–1.06)	1.03 (1.01–1.05)	1.07 (1.03–1.10)	1.04 (1.02–1.07)
Tibiofemoral	1.17 (0.67–2.04)	1.10 (1.06–1.15)	1.01 (0.97–1.06)	1.03 (1.01–1.05)	1.06 (1.03–1.10)	1.04 (1.02–1.07)
Patellofemoral	1.41 (1.10–1.81)	1.05 (1.02–1.08)	1.02 (1.00–1.03)	1.02 (1.01–1.03)	1.02 (1.01–1.04)	1.03 (1.01–1.04)
Any	1.36 (1.07–1.72)	1.06 (1.03–1.09)	1.02 (1.00–1.03)	1.02 (1.01–1.03)	1.03 (1.02–1.04)	1.03 (1.02–1.04)
**Meniscal tear**						
Med. body horizontal∗	0.76 (0.22–2.54)	1.10 (0.98–1.24)	1.02 (0.94–1.09)	1.04 (1.00–1.08)	1.05 (0.98–1.13)	1.05 (1.00–1.11)
Med. posterior horizontal∗	0.76 (0.22–2.54)	1.06 (0.93–1.22)	1.01 (0.93–1.08)	1.02 (0.98–1.07)	1.04 (0.96–1.12)	1.03 (0.98–1.10)
**Other**						
Patellar tend. signal∗	1.29 (0.38–4.37)	1.04 (0.90–1.21)	1.06 (0.98–1.14)	1.03 (0.99–1.07)	1.03 (0.96–1.11)	1.02 (0.95–1.09)
Any ganglion cyst∗	1.24 (0.69–2.22)	1.08 (1.00–1.17)	0.99 (0.96–0.03)	1.02 (1.00–1.04)	1.05 (1.01–1.09)	1.03 (1.00–1.07)
Inf.pat. bursa signal∗	0.82 (0.44–1.53)	1.02 (0.94–1.11)	1.02 (0.99–1.06)	1.02 (0.99–1.04)	1.01 (0.97–1.06)	1.02 (0.98–1.06)
Prepat. bursa signal∗	1.30 (0.78–2.17)	1.05 (0.98–1.13)	0.99 (0.96–0.02)	1.01 (0.99–1.03)	1.02 (0.98–1.05)	1.01 (0.98–1.05)
Joint effusion	1.57 (1.17–2.11)	1.05 (1.02–1.07)	1.02 (1.00–1.04)	1.02 (1.01–1.03)	1.02 (1.01–1.04)	1.03 (1.01–1.04)
Popliteal cyst∗	0.77 (0.47–1.25)	1.04 (0.97–1.12)	1.01 (0.98–1.04)	1.02 (0.99–1.04)	1.02 (0.99–1.06)	1.02 (0.99–1.05)

BML; Bone Marrow Lesion, BMI; Body Mass Index, FT; Full thickness, cx; circumference.

∗ Indicates a logistic regression model and Odds Ratio (OR). Unmarked parameters were analyzed with Poisson regression and the result is given as Relative Risk Ratio (RR).

In unadjusted regression analyses, laboratory parameters at 16 years of age, sex of the participant or smoking status were not notably associated with knee MRI findings (Table S5).

### 3.4 Unadjusted associations of the change in the clinical parameter from 16 to 33 years of age with structural knee MRI findings at 33 years of age

Unadjusted associations of the Δparameters with structural knee MRI findings at 33 years of age are presented in Tables 3 and S6. In contrast with the associations detected for anthropometrics at the age of 16, the Δparameters were not as prominently associated with the knee MRI findings at the age of 33, although some associations with individual knee MRI findings were observed (Table 3 and S6).

For laboratory and other clinical parameters, ΔfP-glucose was positively associated with femoral lateral cartilage loss and all osteophytes. Similarly, ΔCRP was associated with tibiofemoral osteophytes and femoral medial BMLs. The most notable Δparameter associated with knee MRI findings was ΔSBP, being associated with most articular cartilage losses, full thickness cartilage losses, BMLs, and to a smaller extent with osteophytes (Table 3).

**Table 3:** Unadjusted Relative Risks or Odds Ratios with 95% Confidence Intervals of the change in individual clinical parameters from age 16 to 33 for knee MRI findings at age 33.

		ΔBMI(kg/m2)	ΔHeight (cm)	ΔWeight (cm)	ΔfP-glucose (umol/L)	ΔCRP (mg/L)	ΔSystolic bp mmHg)
Cartilage loss	Tibial medial	1.186 (1.076–1.308)	0.896 (0.783–1.026)	1.064 (1.025–1.105)	2.092 (0.964–4.540)	1.050 (0.951–1.160)	1.033 (1.006–1.061)
	Tibial lateral	1.029 (0.911–1.162)	0.950 (0.844–1.070)	1.008 (0.969–1.049)	1.095 (0.580–2.066)	1.037 (0.937–1.148)	1.024 (1.004–1.043)
	Femoral medial	1.049 (0.972–1.132)	0.893 (0.792–1.007)	1.010 (0.983–1.037)	1.473 (0.832–2.608)	0.976 (0.850–1.122)	1.012 (1.000–1.026)
	Femoral lateral	1.069 (0.954–1.197)	0.970 (0.893–1.055)	1.025 (0.985–1.068)	2.170 (1.242–3.792)	1.019 (0.927–1.120)	1.043 (1.029–1.057)
	Tibiofemoral	1.030 (0.969–1.096)	0.907 (0.828–0.993)	1.004 (0.983–1.025)	1.241 (0.743–2.071)	0.967 (0.863–1.084)	1.014 (1.003–1.025)
	Patellofemoral	0.994 (0.958–1.031)	1.001 (0.978–1.024)	0.999 (0.987–1.012)	1.068 (0.834–1.366)	0.968 (0.912–1.028)	1.006 (0.998–1.014)
	Any	0.994 (0.964–1.026)	0.997 (0.974–1.019)	0.999 (0.988–1.009)	1.151 (0.936–1.414)	0.960 (0.910–1.013)	1.007 (1.000–1.013)
FT cartilage loss	Tibial lateral∗	0.996 (0.782–1.268)	0.988 (0.774–1.259)	1.004 (0.924–1.090)	1.226 (0.228–6.609)	0.917 (0.612–1.374)	1.049 (1.000–1.101)
	Femoral medial	1.153 (0.978–1.358)	0.873 (0.737–1.033)	1.045 (0.976–1.118)	1.031 (0.231–4.608)	1.096 (0.988–1.216)	1.019 (0.998–1.040)
	Femoral lateral	1.128 (0.968–1.314)	1.008 (0.914–1.112)	1.056 (1.001–1.113)	1.514 (0.469–4.891)	0.990 (0.880–1.112)	1.032 (1.005–1.060)
	Tibiofemoral	1.095 (0.974–1.232)	0.975 (0.876–1.085)	1.033 (0.990–1.079)	0.950 (0.381–2.373)	1.056 (0.955–1.168)	1.025 (1.006–1.044)
	Patellofemoral	0.936 (0.795–1.101)	0.982 (0.917–1.052)	0.975 (0.926–1.028)	1.384 (0.688–2.787)	1.040 (0.932–1.160)	1.013 (0.998–1.028)
	Any	0.985 (0.872–1.113)	0.990 (0.939–1.043)	0.995 (0.956–1.036)	1.166 (0.625–2.175)	1.029 (0.941–1.126)	1.016 (1.003–1.029)
Size of BML	Tibial lateral∗	1.024 (0.824–1.272)	0.974 (0.760–1.249)	1.018 (0.945–1.097)	1.661 (0.397–6.944)	0.946 (0.673–1.330)	1.052 (1.003–1.104)
	Femoral medial	1.217 (1.007–1.471)	0.817 (0.651–1.024)	1.066 (0.988–1.150)	1.902 (0.554–6.529)	1.148 (1.033–1.277)	1.026 (1.010–1.044)
	Femoral lateral	1.040 (0.938–1.154)	0.979 (0.882–1.087)	1.025 (0.987–1.064)	1.311 (0.754–2.278)	0.936 (0.810–1.080)	1.043 (1.019–1.068)
	Tibiofemoral	1.152 (1.011–1.311)	0.914 (0.768–1.089)	1.053 (1.006–1.102)	1.855 (0.810–4.249)	1.093 (0.988–1.210)	1.036 (1.020–1.052)
	Patellofemoral	0.959 (0.831–1.108)	1.007 (0.935–1.084)	0.984 (0.939–1.031)	1.250 (0.668–2.341)	1.051 (0.956–1.156)	1.005 (0.988–1.021)
	Any	1.023 (0.915–1.145)	0.995 (0.930–1.066)	1.009 (0.972–1.046)	1.354 (0.783–2.340)	1.051 (0.966–1.144)	1.016 (1.003–1.030)
% that is BML	Tibial lateral∗	1.024 (0.824–1.272)	0.974 (0.760–1.249)	1.018 (0.945–1.097)	1.661 (0.397–6.944)	0.946 (0.673–1.330)	1.052 (1.003–1.104)
	Femoral medial	1.107 (0.882–1.391)	0.850 (0.718–1.007)	1.027 (0.936–1.127)	1.131 (0.313–4.087)	1.077 (0.963–1.203)	1.028 (1.005–1.051)
	Femoral lateral	1.025 (0.915–1.148)	0.973 (0.850–1.113)	1.017 (0.981–1.054)	1.311 (0.754–2.278)	0.943 (0.805–1.106)	1.042 (1.014–1.071)
	Tibiofemoral	1.064 (0.952–1.190)	0.951 (0.833–1.085)	1.025 (0.984–1.069)	1.298 (0.590–2.855)	1.026 (0.939–1.120)	1.037 (1.018–1.056)
	Patellofemoral	0.964 (0.819–1.133)	0.952 (0.865–1.049)	0.981 (0.930–1.036)	1.409 (0.704–2.820)	0.994 (0.879–1.124)	1.008 (0.990–1.026)
	Any	1.004 (0.904–1.116)	0.963 (0.897–1.033)	1.001 (0.965–1.037)	1.361 (0.793–2.335)	0.991 (0.912–1.078)	1.019 (1.004–1.033)
Osteophytes	Tibial medial	1.145 (1.015–1.290)	0.875 (0.766–1.000)	1.045 (0.997–1.096)	2.775 (1.502–5.127)	1.088 (1.011–1.171)	1.016 (0.999–1.033)
	Tibial lateral	0.986 (0.831–1.170)	1.010 (0.895–1.140)	1.008 (0.955–1.064)	2.499 (1.393–4.483)	1.089 (1.016–1.168)	1.016 (0.999–1.032)
	Femoral medial	1.141 (1.011–1.288)	0.900 (0.796–1.018)	1.049 (1.002–1.098)	2.343 (1.228–4.468)	1.085 (1.007–1.169)	1.026 (1.008–1.043)
	Femoral lateral	1.015 (0.902–1.142)	1.008 (0.932–1.089)	1.011 (0.972–1.051)	1.956 (1.156–3.310)	1.068 (1.006–1.133)	1.007 (0.992–1.022)
	Tibiofemoral	1.004 (0.912–1.105)	0.999 (0.926–1.077)	1.006 (0.974–1.039)	1.872 (1.114–3.146)	1.056 (1.001–1.115)	1.006 (0.993–1.019)
	Patellofemoral	0.995 (0.955–1.036)	0.994 (0.963–1.026)	1.000 (0.987–1.014)	1.438 (1.086–1.905)	0.943 (0.887–1.001)	1.011 (1.004–1.018)
	Any	0.986 (0.950–1.023)	1.007 (0.988–1.026)	0.999 (0.987–1.012)	1.430 (1.097–1.866)	0.969 (0.919–1.021)	1.009 (1.003–1.016)
Meniscal tear	Med. body horizontal∗	1.134 (0.970–1.325)	0.791 (0.611–1.024)	1.036 (0.978–1.097)	1.387 (0.371–5.180)	1.017 (0.841–1.228)	1.023 (0.987–1.060)
	Med. posterior horizontal∗	1.012 (0.853–1.201)	0.866 (0.665–1.129)	0.996 (0.938–1.057)	2.073 (0.645–6.662)	0.999 (0.811–1.230)	1.007 (0.971–1.044)
Other	Patellar tend. signal∗	1.066 (0.905–1.257)	0.894 (0.688–1.160)	1.019 (0.961–1.080)	1.802 (0.564–5.755)	1.079 (0.935–1.246)	1.020 (0.986–1.056)
	Any ganglion cyst ∗	0.928 (0.853–1.011)	0.806 (0.691–0.939)	0.960 (0.932–0.990)	0.596 (0.323–1.101)	1.005 (0.911–1.109)	0.995 (0.978–1.012)
	Inf.pat. bursa signal∗	0.984 (0.900–1.076)	1.000 (0.923–1.082)	0.995 (0.966–1.026)	0.798 (0.429–1.487)	1.032 (0.935–1.138)	1.016 (0.998–1.035)
	Prepat. bursa signal∗	1.020 (0.951–1.094)	1.036 (0.974–1.102)	1.013 (0.989–1.038)	1.162 (0.683–1.974)	1.050 (0.965–1.142)	1.010 (0.995–1.026)
	Joint effusion	1.050 (1.007–1.095)	1.012 (0.995–1.030)	1.021 (1.006–1.036)	1.194 (0.883–1.614)	0.951 (0.892–1.013)	1.017 (1.008–1.025)
	Popliteal cyst∗	0.993 (0.928–1.063)	0.960 (0.892–1.033)	0.992 (0.969–1.015)	1.013 (0.620–1.655)	0.937 (0.849–1.035)	1.013 (0.998–1.028)

BML; Bone Marrow Lesion, BMI; Body Mass Index, FT; Full thickness, fP-; fasted plasma, hs-CRP; high-sensitivity C-Reactive Protein, bp; blood pressure.

∗ Indicates a logistic regression model and Odds Ratio (OR).

### 3.5 Adjusted associations of BMI at 16 years of with structural knee MRI findings at 33 years of age

Based on the unadjusted associations, selected parameters were evaluated in further adjusted regression models (Table 4, Table 5, Table 6). As anthropometrics are strongly influenced by sex, we evaluated the association of BMI at 16 years of age in a sex-adjusted model (Model 1, Table 4). Most of the associations with knee MRI findings at 16 years of age were not notably influenced by sex.

**Table 4:** Adjusted Relative Risks or Odds Ratios with 95% Confidence Intervals for knee MRI findings at age 33 derived from multivariable regression models including all parameters presented for each model.

		Model 1		Model 2		
		BMI at age 16 (kg/m^2^)	Male sex	BMI at age 16 (kg/m^2^)	Male sex	BMI at age 33 (kg/m^2^)
Cartilage loss	Tibial medial	1.113 (1.048–1.183)	0.757 (0.248–2.312)	1.006 (0.938–1.080)	1.021 (0.359–2.905)	1.218 (1.126–1.316)
	Tibial lateral	1.101 (1.052–1.152)	1.612 (0.745–3.490)	1.054 (0.992–1.120)	1.709 (0.821–3.558)	1.085 (0.992–1.187)
	Femoral medial	1.079 (1.032–1.127)	0.935 (0.544–1.609)	1.032 (0.977–1.090)	0.977 (0.574–1.662)	1.077 (1.017–1.140)
	Femoral lateral	1.084 (1.018–1.153)	2.322 (0.853–6.318)	1.019 (0.950–1.092)	2.537 (1.045–6.159)	1.114 (0.996–1.245)
	Tibiofemoral	1.089 (1.044–1.136)	1.201 (0.715–2.018)	1.031 (0.982–1.082)	1.292 (0.798–2.092)	1.101 (1.038–1.168)
	Patellofemoral	1.027 (1.001–1.054)	0.982 (0.751–1.283)	1.026 (0.992–1.060)	0.982 (0.751–1.283)	1.002 (0.967–1.038)
	Any	1.027 (1.005–1.050)	1.053 (0.837–1.324)	1.026 (0.997–1.055)	1.053 (0.837–1.324)	1.003 (0.972–1.034)
FT cartilage loss	Tibial lateral∗	1.044 (0.848–1.284)	0.165 (0.018–1.498)	1.035 (0.787–1.361)	0.164 (0.018–1.496)	1.013 (0.766–1.338)
	Femoral medial	1.101 (1.005–1.206)	0.669 (0.148–3.014)	1.008 (0.911–1.115)	0.810 (0.171–3.844)	1.172 (1.000–1.374)
	Femoral lateral	1.083 (0.996–1.177)	2.238 (0.480–10.429)	0.985 (0.897–1.081)	2.695 (0.745–9.740)	1.173 (1.013–1.357)
	Tibiofemoral	1.082 (1.005–1.165)	1.367 (0.489–3.819)	1.009 (0.930–1.095)	1.517 (0.547–4.207)	1.126 (1.006–1.259)
	Patellofemoral	1.104 (1.053–1.158)	1.233 (0.626–2.429)	1.104 (1.016–1.199)	1.234 (0.634–2.400)	1.001 (0.868–1.154)
	Any	1.085 (1.037–1.135)	1.344 (0.750–2.411)	1.068 (0.999–1.142)	1.359 (0.762–2.424)	1.029 (0.933–1.136)
Size of BML	Tibial lateral∗	1.071 (0.903–1.270)	0.129 (0.015–1.122)	1.028 (0.812–1.302)	0.124 (0.014–1.097)	1.070 (0.839–1.364)
	Femoral medial	1.126 (1.038–1.221)	0.763 (0.125–4.659)	1.008 (0.905–1.123)	1.169 (0.241–5.674)	1.265 (1.049–1.525)
	Femoral lateral	1.066 (0.966–1.175)	2.963 (0.494–17.778)	1.019 (0.897–1.159)	3.085 (0.542–17.555)	1.076 (0.988–1.171)
	Tibiofemoral	1.104 (1.032–1.180)	1.507 (0.518–4.386)	1.001 (0.922–1.086)	1.931 (0.755–4.942)	1.198 (1.066–1.346)
	Patellofemoral	1.074 (1.016–1.135)	1.312 (0.595–2.893)	1.078 (0.989–1.175)	1.310 (0.599–2.865)	0.994 (0.862–1.146)
	Any	1.074 (1.026–1.125)	1.549 (0.829–2.893)	1.040 (0.976–1.108)	1.590 (0.875–2.892)	1.057 (0.955–1.170)
% that is BML	Tibial lateral∗	1.071 (0.903–1.270)	0.129 (0.015–1.122)	1.028 (0.812–1.302)	0.124 (0.014–1.097)	1.070 (0.839–1.364)
	Femoral medial	1.117 (1.025–1.217)	0.828 (0.155–4.417)	1.038 (0.916–1.177)	0.977 (0.183–5.208)	1.155 (0.976–1.367)
	Femoral lateral	1.087 (1.015–1.165)	2.242 (0.386–13.038)	1.046 (0.956–1.143)	2.341 (0.431–12.698)	1.073 (0.980–1.175)
	Tibiofemoral	1.095 (1.026–1.168)	1.800 (0.672–4.824)	1.032 (0.947–1.125)	1.972 (0.735–5.287)	1.113 (1.014–1.221)
	Patellofemoral	1.094 (1.036–1.155)	0.988 (0.451–2.164)	1.085 (0.992–1.186)	0.993 (0.455–2.167)	1.016 (0.903–1.142)
	Any	1.085 (1.041–1.132)	1.360 (0.749–2.470)	1.058 (0.993–1.127)	1.389 (0.764–2.527)	1.046 (0.969–1.130)
Osteophytes	Tibial medial	1.117 (1.055–1.182)	1.167 (0.391–3.482)	1.020 (0.954–1.091)	1.499 (0.550–4.080)	1.196 (1.086–1.317)
	Tibial lateral	1.142 (1.093–1.192)	1.234 (0.491–3.103)	1.092 (1.025–1.163)	1.357 (0.550–3.345)	1.102 (0.988–1.230)
	Femoral medial	1.107 (1.043–1.175)	1.509 (0.541–4.213)	1.009 (0.943–1.080)	1.908 (0.762–4.774)	1.191 (1.068–1.327)
	Femoral lateral	1.104 (1.060–1.150)	1.043 (0.533–2.044)	1.065 (1.006–1.126)	1.091 (0.565–2.106)	1.071 (0.981–1.169)
	Tibiofemoral	1.104 (1.060–1.150)	1.177 (0.656–2.112)	1.069 (1.014–1.127)	1.222 (0.694–2.153)	1.063 (0.987–1.145)
	Patellofemoral	1.051 (1.019–1.085)	1.398 (1.077–1.815)	1.042 (1.003–1.083)	1.402 (1.082–1.815)	1.014 (0.974–1.057)
	Any	1.061 (1.034–1.088)	1.341 (1.052–1.710)	1.054 (1.020–1.088)	1.344 (1.057–1.710)	1.011 (0.974–1.049)
Meniscal tear	Med. body horizontal∗	1.098 (0.975–1.236)	1.003 (0.274–3.675)	1.003 (0.851–1.182)	0.868 (0.228–3.301)	1.161 (1.003–1.344)
	Med. posterior horizontal∗	1.065 (0.932–1.216)	1.015 (0.278–3.701)	1.041 (0.875–1.239)	1.005 (0.275–3.673)	1.035 (0.880–1.218)
Other	Patellar tend. signal∗	1.040 (0.897–1.206)	1.024 (0.281–3.728)	0.987 (0.808–1.207)	0.994 (0.271–3.651)	1.075 (0.918–1.259)
	Any ganglion cyst ∗	1.081 (1.001–1.168)	1.471 (0.779–2.778)	1.122 (1.015–1.240)	1.470 (0.777–2.782)	0.951 (0.873–1.036)
	Inf.pat. bursa signal∗	1.026 (0.942–1.116)	0.790 (0.410–1.521)	1.033 (0.928–1.150)	0.790 (0.410–1.521)	0.990 (0.903–1.085)
	Prepat. bursa signal∗	1.051 (0.979–1.128)	1.275 (0.748–2.174)	1.025 (0.938–1.119)	1.274 (0.747–2.173)	1.034 (0.963–1.110)
	Joint effusion	1.051 (1.027–1.077)	1.632 (1.200–2.219)	1.006 (0.976–1.037)	1.685 (1.252–2.268)	1.070 (1.030–1.111)
	Popliteal cyst∗	1.046 (0.976–1.122)	0.787 (0.473–1.310)	1.043 (0.955–1.139)	0.787 (0.473–1.310)	1.004 (0.936–1.077)

BML; Bone Marrow Lesion, BMI; Body Mass Index, FT; Full thickness, fP-; fasted plasma, hs-CRP; high-sensitivity C-Reactive Protein, bp; blood pressure.

∗ Indicates a logistic regression model and Odds Ratio (OR).

**Table 5:** Adjusted Relative Risks or Odds Ratios with 95% Confidence Intervals for knee MRI findings at age 33 derived from multivariable regression models including all parameters presented for each model, and sex as covariates.

		Model 1		Model 1		Model 1	
		BMI at age 16 (kg/m^2^)	ΔBMI(kg/m2)	BMI at age 16 (kg/m^2^)	ΔfP-glucose (umol/L)	BMI at age 16 (kg/m^2^)	ΔSystolic bp mmHg)
Cartilage loss	Tibial medial	1.225 (1.123–1.337)	1.218 (1.126–1.316)	1.108 (1.035–1.186)	2.221 (0.894–5.517)	1.083 (1.023–1.147)	1.041 (1.008–1.076)
	Tibial lateral	1.143 (1.063–1.230)	1.085 (0.992–1.187)	1.097 (1.046–1.149)	1.013 (0.499–2.056)	1.083 (1.035–1.134)	1.017 (0.996–1.039)
	Femoral medial	1.111 (1.055–1.171)	1.077 (1.017–1.140)	1.081 (1.033–1.130)	1.479 (0.790–2.769)	1.076 (1.028–1.126)	1.011 (0.998–1.025)
	Femoral lateral	1.135 (1.033–1.247)	1.114 (0.996–1.245)	1.085 (1.013–1.162)	1.918 (0.947–3.885)	1.049 (0.990–1.112)	1.036 (1.018–1.055)
	Tibiofemoral	1.135 (1.076–1.197)	1.101 (1.038–1.168)	1.088 (1.042–1.135)	1.508 (0.909–2.504)	1.074 (1.031–1.119)	1.021 (1.008–1.034)
	Patellofemoral	1.028 (0.998–1.058)	1.002 (0.967–1.038)	1.034 (1.009–1.059)	1.049 (0.818–1.343)	1.027 (1.000–1.054)	1.007 (0.998–1.017)
	Any	1.028 (1.003–1.055)	1.003 (0.972–1.034)	1.031 (1.009–1.053)	1.120 (0.905–1.387)	1.025 (1.002–1.049)	1.007 (1.000–1.015)
FT cartilage loss	Tibial lateral∗	1.049 (0.830–1.325)	1.013 (0.766–1.338)	1.042 (0.849–1.279)	0.974 (0.171–5.559)	1.001 (0.779–1.287)	1.049 (0.986–1.116)
	Femoral medial	1.181 (0.992–1.407)	1.172 (1.000–1.374)	1.104 (1.003–1.214)	1.125 (0.216–5.865)	1.092 (0.993–1.200)	1.034 (1.011–1.057)
	Femoral lateral	1.155 (0.996–1.339)	1.173 (1.013–1.357)	1.083 (0.973–1.205)	1.279 (0.297–5.502)	1.058 (0.983–1.139)	1.023 (0.987–1.061)
	Tibiofemoral	1.135 (1.012–1.274)	1.126 (1.006–1.259)	1.078 (0.996–1.167)	0.894 (0.349–2.287)	1.069 (0.993–1.151)	1.027 (1.004–1.051)
	Patellofemoral	1.105 (1.011–1.207)	1.001 (0.868–1.154)	1.107 (1.055–1.162)	1.347 (0.633–2.866)	1.100 (1.048–1.154)	1.004 (0.987–1.022)
	Any	1.099 (1.025–1.178)	1.029 (0.933–1.136)	1.086 (1.037–1.137)	1.101 (0.570–2.125)	1.079 (1.029–1.132)	1.011 (0.996–1.026)
Size of BML	Tibial lateral∗	1.100 (0.898–1.349)	1.070 (0.839–1.364)	1.069 (0.900–1.268)	1.314 (0.300–5.759)	1.045 (0.863–1.266)	1.035 (0.981–1.093)
	Femoral medial	1.275 (1.050–1.549)	1.265 (1.049–1.525)	1.126 (1.025–1.238)	2.111 (0.419–10.645)	1.124 (1.040–1.216)	1.047 (1.024–1.070)
	Femoral lateral	1.097 (0.981–1.226)	1.076 (0.988–1.171)	1.099 (1.018–1.187)	0.932 (0.461–1.885)	1.030 (0.925–1.146)	1.034 (1.008–1.061)
	Tibiofemoral	1.199 (1.070–1.343)	1.198 (1.066–1.346)	1.113 (1.035–1.197)	1.814 (0.660–4.987)	1.089 (1.019–1.164)	1.038 (1.018–1.059)
	Patellofemoral	1.071 (0.978–1.173)	0.994 (0.862–1.146)	1.078 (1.022–1.137)	1.181 (0.594–2.348)	1.084 (1.030–1.140)	0.999 (0.981–1.018)
	Any	1.099 (1.020–1.184)	1.057 (0.955–1.170)	1.079 (1.030–1.130)	1.256 (0.686–2.301)	1.076 (1.029–1.125)	1.013 (0.997–1.028)
% that is BML	Tibial lateral∗	1.100 (0.898–1.349)	1.070 (0.839–1.364)	1.069 (0.900–1.268)	1.314 (0.300–5.759)	1.045 (0.863–1.266)	1.035 (0.981–1.093)
	Femoral medial	1.199 (1.035–1.389)	1.155 (0.976–1.367)	1.112 (1.017–1.216)	1.174 (0.289–4.775)	1.109 (1.015–1.211)	1.043 (1.020–1.067)
	Femoral lateral	1.122 (1.033–1.218)	1.073 (0.980–1.175)	1.099 (1.018–1.187)	0.932 (0.461–1.885)	1.054 (0.981–1.132)	1.036 (1.005–1.067)
	Tibiofemoral	1.149 (1.056–1.250)	1.113 (1.014–1.221)	1.095 (1.020–1.176)	1.164 (0.516–2.629)	1.077 (1.008–1.151)	1.036 (1.014–1.058)
	Patellofemoral	1.102 (1.024–1.185)	1.016 (0.903–1.142)	1.102 (1.045–1.161)	1.398 (0.675–2.896)	1.090 (1.033–1.149)	1.003 (0.983–1.024)
	Any	1.107 (1.047–1.169)	1.046 (0.969–1.130)	1.090 (1.045–1.137)	1.276 (0.727–2.241)	1.076 (1.030–1.124)	1.015 (0.999–1.032)
Osteophytes	Tibial medial	1.220 (1.119–1.331)	1.196 (1.086–1.317)	1.123 (1.057–1.193)	2.945 (1.565–5.541)	1.103 (1.042–1.167)	1.009 (0.987–1.030)
	Tibial lateral	1.203 (1.116–1.297)	1.102 (0.988–1.230)	1.153 (1.104–1.205)	2.758 (1.591–4.780)	1.129 (1.081–1.179)	1.008 (0.987–1.030)
	Femoral medial	1.201 (1.088–1.327)	1.191 (1.068–1.327)	1.115 (1.046–1.190)	2.378 (1.142–4.950)	1.088 (1.025–1.155)	1.016 (0.996–1.038)
	Femoral lateral	1.140 (1.071–1.213)	1.071 (0.981–1.169)	1.109 (1.063–1.157)	1.994 (1.197–3.320)	1.098 (1.054–1.145)	0.998 (0.981–1.016)
	Tibiofemoral	1.136 (1.075–1.200)	1.063 (0.987–1.145)	1.110 (1.064–1.157)	1.872 (1.127–3.110)	1.099 (1.054–1.145)	0.997 (0.982–1.011)
	Patellofemoral	1.057 (1.020–1.095)	1.014 (0.974–1.057)	1.051 (1.019–1.084)	1.364 (1.038–1.793)	1.045 (1.013–1.079)	1.005 (0.998–1.013)
	Any	1.066 (1.035–1.098)	1.011 (0.974–1.049)	1.062 (1.036–1.088)	1.358 (1.052–1.752)	1.056 (1.029–1.083)	1.003 (0.996–1.010)
Meniscal tear	Med. body horizontal∗	1.165 (1.005–1.350)	1.161 (1.003–1.344)	1.106 (0.976–1.253)	1.417 (0.365–5.501)	1.090 (0.964–1.233)	1.019 (0.977–1.063)
	Med. posterior horizontal∗	1.078 (0.929–1.251)	1.035 (0.880–1.218)	1.068 (0.922–1.238)	2.160 (0.646–7.221)	1.069 (0.932–1.226)	0.999 (0.958–1.043)
Other	Patellar tend. signal∗	1.062 (0.899–1.254)	1.075 (0.918–1.259)	1.026 (0.869–1.212)	1.933 (0.583–6.412)	1.046 (0.907–1.207)	1.025 (0.983–1.070)
	Any ganglion cyst ∗	1.067 (0.983–1.157)	0.951 (0.873–1.036)	1.085 (1.003–1.175)	0.602 (0.321–1.128)	1.076 (0.994–1.166)	0.997 (0.977–1.017)
	Inf.pat. bursa signal∗	1.023 (0.936–1.117)	0.990 (0.903–1.085)	1.041 (0.957–1.133)	0.752 (0.402–1.408)	1.010 (0.925–1.103)	1.019 (0.997–1.042)
	Prepat. bursa signal∗	1.059 (0.985–1.139)	1.034 (0.963–1.110)	1.053 (0.979–1.133)	1.179 (0.689–2.018)	1.041 (0.966–1.122)	1.014 (0.996–1.032)
	Joint effusion	1.076 (1.046–1.108)	1.070 (1.030–1.111)	1.045 (1.021–1.070)	1.103 (0.826–1.473)	1.044 (1.019–1.070)	1.011 (1.002–1.020)
	Popliteal cyst∗	1.047 (0.975–1.126)	1.004 (0.936–1.077)	1.041 (0.970–1.118)	0.971 (0.591–1.597)	1.045 (0.970–1.125)	1.010 (0.992–1.027)

BML; Bone Marrow Lesion, BMI; Body Mass Index, FT; Full thickness, fP-; fasted plasma, hs-CRP; high-sensitivity C-Reactive Protein, bp; blood pressure.

∗ Indicates a logistic regression model and Odds Ratio (OR).

**Table 6:** Adjusted Relative Risks or Odds Ratios with 95% Confidence Intervals for knee MRI findings at age 33 derived from multivariable regression models including all parameters presented for each model, and sex as covariates.

		Model 3			Model 3		
		BMI at age 16 (kg/m^2^)	ΔfP-glucose (umol/L)	fP-glucose at age 16 (umol/L)	BMI at age 16 (kg/m^2^)	ΔSystolic bp (mmHg)	Systolic bp at age 16 mmHg)
Cartilage loss	Tibial medial	1.103 (1.031–1.181)	3.818 (1.235–11.800)	1.793 (1.061–3.032)	1.080 (1.019–1.145)	1.047 (1.019–1.076)	1.016 (0.952–1.085)
	Tibial lateral	1.096 (1.045–1.148)	1.217 (0.407–3.639)	1.165 (0.738–1.838)	1.079 (1.027–1.134)	1.032 (1.009–1.055)	1.032 (0.988–1.077)
	Femoral medial	1.079 (1.032–1.129)	1.636 (0.721–3.713)	1.132 (0.724–1.772)	1.076 (1.029–1.125)	1.011 (0.988–1.035)	0.999 (0.964–1.036)
	Femoral lateral	1.082 (1.006–1.163)	2.301 (0.857–6.180)	1.290 (0.468–3.556)	1.051 (0.990–1.115)	1.032 (1.010–1.055)	0.991 (0.943–1.041)
	Tibiofemoral	1.086 (1.041–1.133)	1.828 (0.909–3.674)	1.254 (0.852–1.845)	1.073 (1.030–1.117)	1.025 (1.007–1.042)	1.008 (0.974–1.044)
	Patellofemoral	1.035 (1.010–1.061)	0.926 (0.631–1.358)	0.872 (0.641–1.187)	1.026 (1.000–1.054)	1.007 (0.995–1.020)	1.000 (0.983–1.017)
	Any	1.032 (1.010–1.054)	1.023 (0.738–1.419)	0.899 (0.684–1.181)	1.025 (1.003–1.049)	1.006 (0.996–1.016)	0.998 (0.984–1.012)
FT cartilage loss	Tibial lateral∗	1.041 (0.847–1.280)	1.046 (0.105–10.471)	1.082 (0.215–5.456)	1.002 (0.779–1.289)	1.047 (0.967–1.133)	0.994 (0.880–1.122)
	Femoral medial	1.103 (1.005–1.210)	1.318 (0.101–17.160)	1.137 (0.416–3.108)	1.094 (1.009–1.187)	1.029 (0.968–1.093)	0.987 (0.880–1.107)
	Femoral lateral	1.081 (0.967–1.208)	1.474 (0.391–5.556)	1.194 (0.368–3.871)	1.071 (0.989–1.159)	0.984 (0.914–1.058)	0.919 (0.844–1.001)
	Tibiofemoral	1.078 (0.998–1.165)	0.929 (0.178–4.863)	1.030 (0.524–2.022)	1.075 (1.003–1.154)	1.010 (0.963–1.059)	0.960 (0.888–1.038)
	Patellofemoral	1.119 (1.067–1.173)	0.700 (0.162–3.015)	0.369 (0.066–2.069)	1.100 (1.049–1.154)	1.003 (0.970–1.038)	0.998 (0.948–1.050)
	Any	1.093 (1.043–1.146)	0.696 (0.233–2.081)	0.502 (0.131–1.928)	1.082 (1.036–1.130)	1.002 (0.974–1.032)	0.983 (0.939–1.028)
Size of BML	Tibial lateral∗	1.067 (0.898–1.269)	1.397 (0.180–10.836)	1.092 (0.149–7.987)	1.044 (0.860–1.267)	1.040 (0.969–1.116)	1.010 (0.908–1.124)
	Femoral medial	1.124 (1.022–1.236)	4.338 (0.403–46.659)	1.967 (0.762–5.073)	1.113 (1.034–1.199)	1.064 (1.007–1.124)	1.051 (0.922–1.196)
	Femoral lateral	1.100 (0.980–1.236)	0.869 (0.008–99.369)	NA	1.054 (0.931–1.195)	0.972 (0.918–1.030)	0.874 (0.782–0.977)
	Tibiofemoral	1.110 (1.036–1.190)	2.308 (0.465–11.458)	1.362 (0.561–3.306)	1.089 (1.023–1.159)	1.038 (1.000–1.078)	1.000 (0.925–1.080)
	Patellofemoral	1.086 (1.028–1.148)	0.725 (0.182–2.883)	0.476 (0.084–2.693)	1.083 (1.028–1.140)	1.003 (0.965–1.043)	1.008 (0.956–1.061)
	Any	1.086 (1.036–1.137)	0.836 (0.250–2.792)	0.537 (0.131–2.197)	1.076 (1.030–1.123)	1.012 (0.982–1.042)	0.999 (0.956–1.043)
% that is BML	Tibial lateral∗	1.067 (0.898–1.269)	1.397 (0.180–10.836)	1.092 (0.149–7.987)	1.044 (0.860–1.267)	1.040 (0.969–1.116)	1.010 (0.908–1.124)
	Femoral medial	1.111 (1.016–1.215)	1.737 (0.297–10.145)	1.351 (0.679–2.688)	1.114 (1.030–1.205)	1.035 (0.982–1.091)	0.979 (0.889–1.079)
	Femoral lateral	1.100 (0.980–1.236)	0.869 (0.008–99.369)	NA	1.086 (0.986–1.195)	0.961 (0.901–1.025)	0.848 (0.759–0.948)
	Tibiofemoral	1.096 (1.021–1.177)	1.084 (0.340–3.450)	0.907 (0.307–2.680)	1.084 (1.013–1.159)	1.021 (0.988–1.055)	0.963 (0.903–1.028)
	Patellofemoral	1.111 (1.052–1.173)	0.812 (0.249–2.648)	0.440 (0.079–2.451)	1.089 (1.030–1.150)	1.007 (0.972–1.043)	1.007 (0.960–1.056)
	Any	1.099 (1.052–1.148)	0.753 (0.321–1.766)	0.445 (0.130–1.523)	1.078 (1.034–1.124)	1.009 (0.983–1.036)	0.988 (0.950–1.028)
Osteophytes	Tibial medial	1.117 (1.050–1.188)	4.116 (1.634–10.367)	1.555 (0.696–3.474)	1.101 (1.041–1.164)	1.014 (0.978–1.052)	1.012 (0.957–1.070)
	Tibial lateral	1.157 (1.106–1.210)	2.212 (0.612–7.991)	0.738 (0.162–3.370)	1.131 (1.081–1.183)	1.003 (0.968–1.040)	0.989 (0.937–1.045)
	Femoral medial	1.111 (1.042–1.184)	3.256 (1.196–8.863)	1.512 (0.786–2.908)	1.089 (1.029–1.154)	1.012 (0.974–1.052)	0.991 (0.934–1.051)
	Femoral lateral	1.114 (1.065–1.165)	1.531 (0.606–3.865)	0.684 (0.219–2.131)	1.097 (1.053–1.143)	1.002 (0.972–1.032)	1.007 (0.967–1.048)
	Tibiofemoral	1.111 (1.063–1.160)	1.764 (0.807–3.857)	0.917 (0.378–2.220)	1.101 (1.056–1.147)	0.991 (0.965–1.018)	0.989 (0.954–1.026)
	Patellofemoral	1.049 (1.017–1.082)	1.655 (1.238–2.211)	1.233 (1.065–1.427)	1.046 (1.013–1.079)	1.005 (0.994–1.016)	0.999 (0.982–1.015)
	Any	1.060 (1.034–1.086)	1.650 (1.254–2.171)	1.234 (1.083–1.407)	1.056 (1.030–1.083)	1.002 (0.991–1.013)	0.998 (0.982–1.014)
Meniscal tear	Med. body horizontal∗	1.109 (0.977–1.259)	1.144 (0.159–8.217)	0.730 (0.082–6.492)	1.097 (0.968–1.243)	0.999 (0.937–1.065)	0.958 (0.873–1.053)
	Med. posterior horizontal∗	1.070 (0.921–1.242)	1.957 (0.296–12.930)	0.868 (0.106–7.078)	1.070 (0.932–1.228)	0.995 (0.932–1.061)	0.991 (0.907–1.083)
Other	Patellar tend. signal∗	1.030 (0.871–1.217)	1.512 (0.240–9.514)	0.701 (0.090–5.478)	1.047 (0.907–1.208)	1.022 (0.965–1.083)	0.993 (0.911–1.082)
	Any ganglion cyst ∗	1.083 (1.000–1.172)	0.752 (0.304–1.863)	1.217 (0.651–2.275)	1.075 (0.992–1.165)	1.010 (0.981–1.040)	1.025 (0.985–1.068)
	Inf.pat. bursa signal∗	1.056 (0.969–1.151)	0.260 (0.088–0.768)	0.245 (0.074–0.814)	1.009 (0.924–1.102)	1.024 (0.993–1.056)	1.010 (0.967–1.054)
	Prepat. bursa signal∗	1.055 (0.981–1.136)	1.003 (0.468–2.150)	0.837 (0.442–1.586)	1.041 (0.966–1.122)	1.013 (0.987–1.040)	0.999 (0.964–1.036)
	Joint effusion	1.046 (1.022–1.071)	1.038 (0.702–1.536)	0.931 (0.694–1.249)	1.045 (1.019–1.072)	1.004 (0.990–1.018)	0.986 (0.968–1.006)
	Popliteal cyst∗	1.043 (0.971–1.121)	0.829 (0.406–1.693)	0.866 (0.534–1.404)	1.044 (0.970–1.125)	1.013 (0.987–1.039)	1.006 (0.972–1.042)

BML; Bone Marrow Lesion, BMI; Body Mass Index, FT; Full thickness, fP-; fasted plasma, hs-CRP; high-sensitivity C-Reactive Protein, bp; blood pressure.

∗ Indicates a logistic regression model and Odds Ratio (OR).

Subsequently, we adjusted the association also with BMI at age 33 (Model 2, Table 4), as BMI was most notably associated with knee MRI findings cross-sectionally in the 33-year timepoint^8^. Although BMI at 33 years of age was associated with more structural knee MRI findings, BMI at age 16 was notably associated with most osteophytes (Model 2, Table 4).

### 3.6 Adjusted associations of Δparameters with structural knee MRI findings at 33 years of age

Next, we evaluated the associations of Δparameters in adjusted models (Table 5, Table 6). As anthropometrics at age 16 were most notably associated with knee MRI findings at 33 years of age, and differences in the Δparameters were observed according to sex (Tables S1–S2), we adjusted the associations with sex and BMI at 16 years of age (Model 1, Table 5). After these adjustments ΔBMI was associated with notably more knee MRI findings than in the unadjusted model. For, ΔfP-glucose, the association with osteophytes was not notably influenced by these additional adjustments. For ΔSBP, most of the associations observed in the unadjusted model, were nullified by these additional adjustments (Model 1, Table 5).

When we further adjusted these associations with the parameter of interest at 16 years of age, most of the associations with ΔfP-glucose and osteophytes remained, whereas the associations of ΔSBP were nullified entirely (Model 3, Table 6).

### 3.7 Evaluation of clinical parameters according to classes of BMI and ΔfP-glucose

As a validation for the results of the regression analyses, we evaluated the other clinical parameters in classes of BMI at 16 years of age in descriptive statistics (Table S7). In subjects with BMI <25, ΔSBP was negative (−4.8 to −5.7 mmHg), whereas in subjects with BMI >25 ΔSBP was positive (5.4 mmHg). For ΔfP-glucose no apparent difference was observed according to the BMI classes (Table S7). ΔBMI was the smallest (2.9 kg/m^2^) in subjects with BMI <25 (Table S7).

As BMI did not seem to influence ΔfP-glucose, we lastly evaluated the clinical parameters in classes ΔfP-glucose to determine possible explanations for the associations detected in the regression models for ΔfP-glucose (Table S8). Although BMI at age 16 was roughly the same for all classes of ΔfP-glucose, BMI at age 33 was 2.6 kg/m^2^ greater in subjects with ΔfP-glucose >0.3 compared to those with ΔfP-glucose < −0.3 (Table S8). Also, Δwaist cx. (7.7 cm) and Δweight (7.9 kg) were also greater in subjects with ΔfP-glucose >0.3 compared to those with ΔfP-glucose < −0.3 (Table S8).

## 4 Discussion

In this study, we evaluated the potential associations between clinical characteristics in adolescence and knee MRI findings in young adulthood in the NFBC1986 subpopulation. We detected associations of anthropometric parameters at age 16 and the change in laboratory parameters such as fasting glucose and hs-CRP, and clinical parameters such as SBP from age 16 to 33, with MRI findings at age 33. Most of the associations were moderated by body composition, as they were nullified by adjusting for BMI at age 16. Moreover, as ΔBMI was also associated with MRI findings at age 33 only after adjusting for BMI at age 16, our results suggest that the body composition already at adolescence is a determining factor of MRI findings in 33-year-olds.

It is generally thought that OA-associated structural MRI findings such as cartilage lesions and osteophytes are absent in atraumatic adolescent knees [29]. Further circumstantial evidence was provided by a recent large population-based descriptive cohort study describing normal variants and typically encountered abnormalities in 3800 adolescent knee MRIs. Osteophytes, cartilage lesions or meniscal lesions were not mentioned [30]. In our current study, anthropometrics at adolescence were associated with numerous imaging findings in young adulthood already in the initial unadjusted analyses, whereas changes in anthropometrics between the time points were not. These results suggest that body composition already in adolescence is a strong determining factor for knee imaging findings in young adults. As higher body mass is a well-established risk factor for knee OA in middle age [8], our results also suggest that there might exist a bodyweight and/or related anthropometric risk threshold for developing MRI findings. Moreover, as height at age 16 was the only metric not associated with imaging findings at young adulthood, the results would suggest an association with sheer mass rather than body size.

As to why no associations with ΔBMI or other anthropometrics and knee MRI findings were observed in the initial analyses, is likely explained by the smaller ΔBMI in overweight subjects (+2.9 kg/m^2^) from age 16 to 33 compared to other subjects (+4.8 kg/m^2^). Our regression models support this interpretation, as multiple associations of ΔBMI with imaging were observed after adjusting with BMI at age 16. Therefore, we conclude that heavier body composition at adolescence and, to a smaller extent, increase in body mass seem to be associated with knee MRI findings later in life. In the short term, however, adolescent obesity does not seem to cause accumulation of imaging findings; in 2003, Jones et al. evaluated the effects of sex, growth, Tanner stage and physical activity on knee articular cartilage volume development in 74 participants aged 9–18 years. In their results, overweight participants did not differ in their articular cartilage volume at baseline MRI or in short 1–2-year follow-up imaging [31].

Interestingly, clinical parameters at age 16 were not associated with MRI findings at age 33, whereas most Δparameters, most notably ΔSBP and ΔfP-glucose, were. The results are likely to some extent explained by the moderating effect of body composition on these clinical metrics. In the initial analyses, ΔSBP was associated with multiple knee MRI findings, whereas SBP at age 16 (or 33 [8]) was not. This is likely explained by the fact that overweight and ageing, both key risk factors for knee OA, gradually elevate BP and the risk of hypertension [32,33]. Furthermore, the incidence of hypertension in adolescents even with obesity or overweight is relatively low [34] compared to adults [33]. Therefore, it is likely that overweight adolescents do not yet have but are at an increased risk for knee MRI findings as well as hypertension in adulthood. This interpretation is supported by our regression analyses, where the associations of ΔSBP with knee MRI findings were largely mediated by BMI at adolescence and mostly nullified when also accounting for SBP at adolescence. Our findings contrast with a meta-analysis where an association between hypertension and knee OA (both radiographic and symptomatic) was reported [35], but are supported by the findings of a Mendelian randomization (MR) study, where an MR analysis did not uncover any causal relationship between OA and hypertension [36]. Therefore, we conclude that hypertension itself is not associated with knee MRI findings, but rather the association is mediated by higher body mass, hip and waist circumference, which often co-exists with hypertension.

Similarly, as what was observed for ΔSBP in the initial analyses, ΔfP-glucose was associated with knee MRI findings, whereas fP-glucose at age 16 (or 33 [8]) was not. Interestingly, the associations were limited to osteophytes. Increased fasting glucose, a sign of impaired glucose metabolism, is, like with hypertension, associated with obesity [37]. The global prevalence of type 2 diabetes (T2D) is alarmingly increasing also in adolescents, but relatively low in 16-year-olds compared to 33-year-olds [38]. Moreover, over 75% of children with T2D have obesity at diagnosis, and the prevalence of T2D is 1.3% in children with obesity, 13-fold that in children with normal weight (0.1%), according to a meta-analysis of over 200 000 children [39]. Thus, it is likely that subjects with overweight at age 16 are more prone to impaired glucose metabolism and elevated fasting glucose at age 33, suggesting the association of ΔfP-glucose and knee MRI findings could be mediated by body composition. In fact, subjects with ΔfP-glucose >0.3 mmol/l had the highest ΔBMI (+5.9 kg/m^2^) and the highest Δwaist circumference (+17.6 cm) compared to subjects with ΔfP-glucose <0.3 mmol/l (+4.2 kg/m^2^ and +12.8 cm, respectively). However, the associations of ΔfP-glucose with knee MRI findings remained largely unchanged by adjusting for BMI at age 16, supporting the interpretation, that the associations of glucose metabolism with knee MRI findings, are to some extent but not entirely mediated by body composition [40]. Our results are also supported by a meta-analysis, where T2D was associated with the development and presence of radiographic and symptomatic OA even when controlling for BMI [41], and a recent register-based cohort study, where accumulation of metabolic conditions (obesity, hypertension and diabetes) increased patient-reported pain in hip and knee OA [42]. Previous clinical data regarding the specific link between osteophyte formation and glucose metabolism are sparse and based on limited sample sizes and radiographs [43]. In a histological analysis of a murine model of OA, osteophyte formation was reported to be higher in hyperglycemic mice [44]. Based on our results, a direct link between impaired glucose metabolism and osteophyte formation might exist. Thus, mechanistic and causal studies on this matter are warranted.

In our results, identified associations with MOAKS-described MRI findings (cartilage lesion area rather than cartilage thickness is evaluated) were not notably influenced by sex. Cartilage volume in young adults has been proposed as a marker of knee joint health, but the relationship between overall cartilage volume and thickness and OA, compared to cartilage lesions and OA, is less understood [22,[45], [46], [47]]. As a speculation, presuming that evaluated MRI findings are absent in adolescents; our findings suggest that the accumulation of OA-associated structural findings could still be prevented in school health and primary care. In this regard our findings would contrast with the results by Wills et al. where no additional risk for clinically evident knee OA at age of 53 years was seen from childhood and adolescence BMI once adult BMI had been accounted for [48]. A central advantage of our study is the availability of MRI for the study participants in young adulthood as radiographs tend to miss subtle OA findings [49]. Definite knee OA is still rare in this age group and participants are mostly asymptomatic [8], making clinical evaluation limited [50]. We concur that the accumulative effect of lifetime BMI carries the most significant risk for developing knee OA. Planned follow-up of the NFBC1986 subpopulation (including MRI) will provide further knowledge on the effects of lifetime background and clinical characteristics in imaging findings.

Previous knee injuries and especially ACL injuries are an established risk factor of developing knee OA [7,10,11], and some potentially significant knee injury events might not be accounted for. However, clearly substantial injuries have been considered. First, participants were asked about any previous knee injuries in questionnaires. Second, only a few ACL tears and meniscal injuries of potentially traumatic origin were present in MRIs. A clinical evaluation of potential knee malalignment was not available at either time point. Regarding glucose metabolism, we lack some interesting parameters like fasting insulin and parameters from an oral glucose tolerance test at both time points. Moreover, some parameters like physical activity score and alcohol consumption were not comparable at both time points. Although parameters were first analyzed individually and then with multivariable regression models, some residual confounding can exist and the possibility of results being based on chance is higher with a limited sample size. No prior sample size calculations had been made due to limited number of early adulthood knee MRI cohorts and sparse knowledge regarding the frequency of imaging findings. Poisson regression with robust estimates was chosen as the analysis method for reasons stated before^8^. Multiple regression models were evaluated, and the key findings were verified with further descriptive statistics.

In conclusion, of analyzed parameters, higher body mass, hip and waist circumference were most frequently associated with knee MRI findings. Moreover, impaired glucose metabolism could be linked to OA pathogenesis through increased osteophyte formation, regardless of body composition. Although causality is not demonstrated, our results suggest that heavier body composition (and related co-morbidities) significantly contribute to knee OA pathogenesis already from adolescence and are key factors to consider when assessing OA.

## Author contributions

A.K.: Conceptualization, data curation, writing - original draft, writing - review & editing.

J.T.: Conceptualization, formal analysis, visualization, writing - original draft, writing - review & editing.

M.T.Ni: Funding acquisition, resources, writing - review & editing.

S.S.: Funding acquisition, resources, writing - review & editing.

M.T.Ne: Conceptualization, project administration, resources, supervision, writing - review & editing.

The corresponding author (A.K.) takes responsibility for the overall integrity of the research article.

## Role of the funding source

This work was supported by the 10.13039/501100002341Research Council of Finland (Flagship of Advanced Mathematics for Sensing Imaging and Modelling grant #359186 and grant #354692). NFBC1986 33-35y follow-up study received financial support from 10.13039/501100006196University of Oulu (Strategic funding from donations) and Oulu University Hospital (K65760).

## Conflict of interest

The authors declare no conflict of interest.
