# Supplementary material for: Associations of health parameters at 16 years of age with structural knee MRI findings at 33 years of age in a general population-based birth cohort
Source: Osteoarthr Cartil Open. 2026 Mar 14;8(2):100780. doi: 10.1016/j.ocarto.2026.100780 (PMC13049644; doi:10.1016/j.ocarto.2026.100780)
Supplement: Multimedia component 1 [file mmc1.docx]

**Associations of clinical parameters at 16 years of age with structural knee MRI findings at 33 years of age in a general population-based birth cohort**

Antti Kemppainen, MD, PhD^a,b*¤^, Joona Tapio, MD, PhD^c,d¤^, Miika T. Nieminen, PhD^a,b,e^, Simo Saarakkala, PhD^a,b,d,e^, Mika T. Nevalainen, MD, PhD^a,b,d,e^

^a^Research Unit of Health Sciences and Technology

Faculty of Medicine, University of Oulu

P.O. Box 5000, FI-90014 Oulu, Finland

^b^Department of Diagnostic Radiology

Oulu University Hospital

P.O. Box 50, FI-90029 Oulu, Finland

^c^Faculty of Biochemistry and Molecular Medicine, University of Oulu

P.O. Box 5400, FIN-90014 Oulu, Finland

^d^Biocenter Oulu, University of Oulu

P.O. Box 5400, FIN-90014 Oulu, Finland

^e^Medical Research Center Oulu

University of Oulu and Oulu University Hospital

Oulu, Finland

This work was supported by the Research Council of Finland (Flagship of Advanced
Mathematics for Sensing Imaging and Modelling grant #359186 and grant #354692). NFBC1986 33-35y follow-up study received financial support from University of Oulu (Strategic funding from donations) and Oulu University Hospital (K65760).

ORCID iD:

Antti Kemppainen: 0009-0008-8501-8731

Joona Tapio: 0000-0002-9267-1586

Miika T. Nieminen: 0000-0002-2300-2848

Simo Saarakkala: 0000-0003-2850-5484

Mika T. Nevalainen: 0000-0002-9483-7690

*Corresponding author

Antti Kemppainen

Department of Diagnostic Radiology

Oulu University Hospital

P.O. Box 50, FI-90029 Oulu, Finland

E-mail address: antti.kemppainen@pohde.fi

¤ Equal contribution

The authors declare no conflict of interest

**Supplementary data**

**Figure S1. Flow chart representing the study population and analyses done.** NFBC1986: Northern Finland Birth Cohort, MRI; Magnetic Resonance Imaging, OA; osteoarthritis, BML; Bone Marrow Lesion, ACL; Anterior Cruciate Ligament, PCL; Posterior Cruciate Ligament, BMI; Body Mass Index, fP-; fasted plasma, HDL; High-Density Lipoprotein, LDL; Low-Density Lipoprotein**,** hs-CRP: high-sensitivity C-Reactive Protein, Alat; alanine aminotransferase, WOMAC; Western Ontario and McMaster Universities Osteoarthritis Index

**
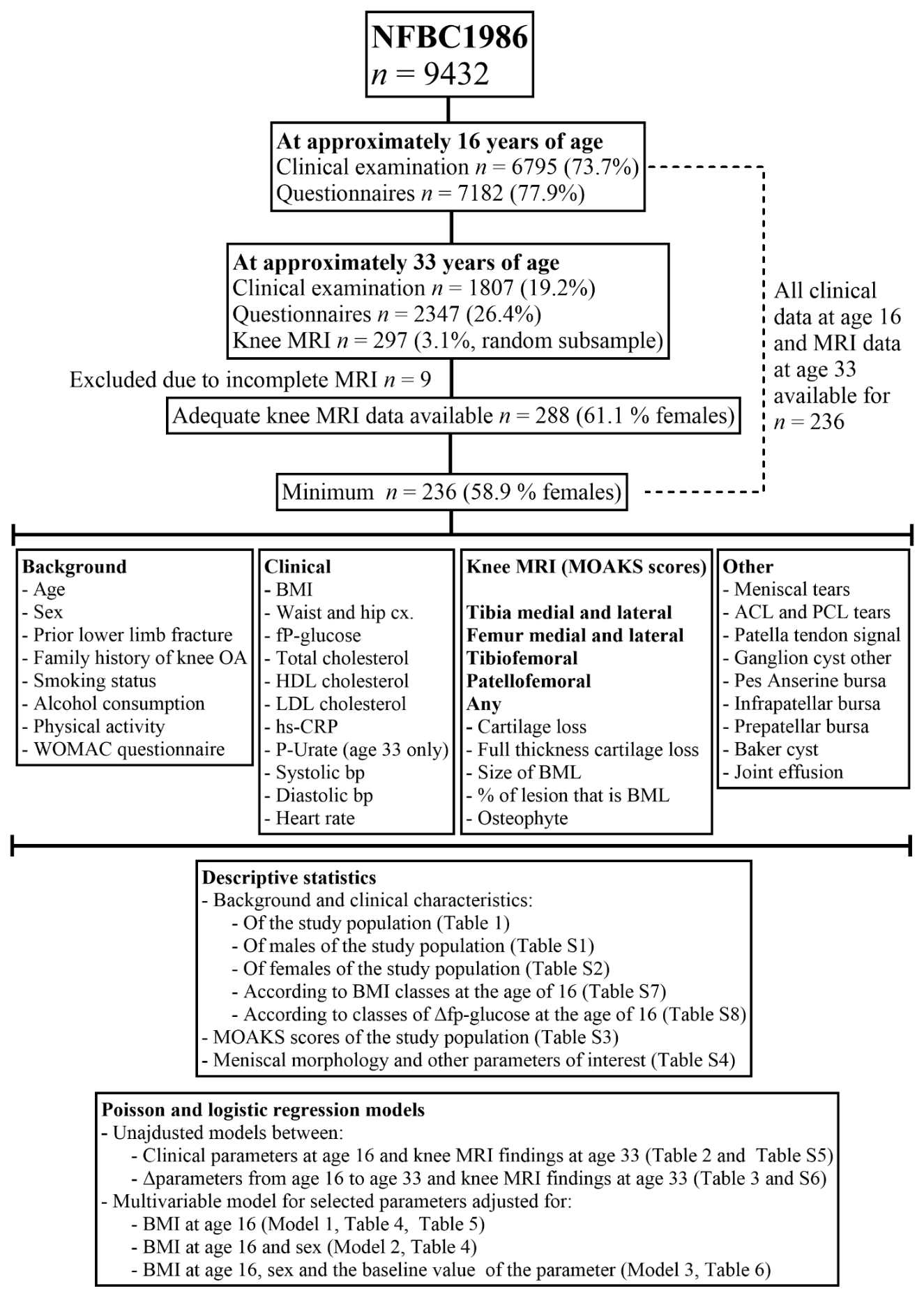
**

**Table S1. Background and clinical characteristics of males of the study population at 16 and 33 years of age and the change in the parameter between the two timepoints.** Data is presented as mean (M) and standard deviation (SD) for normally-distributed continuous variables, median (Mn) and interquartile range (IQR) for skewed continuous variables and count (n) and percentage (%) for count variables. M; Mean, SD; Standard Deviation, OA; osteoarthritis, BMI; Body Mass Index, fP-; fasted plasma, HDL; High-Density Lipoprotein, LDL; Low-Density Lipoprotein, hs-CRP: high-sensitivity C-Reactive Protein.

|  | **At age 16** | | **At age 33** | | **Delta parameter** | |
| --- | --- | --- | --- | --- | --- | --- |
|  | **n** | **M (SD)/Mn(IQR)** | **n** | **M (SD)/Mn(IQR)** | **n** | **M (SD)/Mn(IQR)** |
|  |  | **or n (%)** |  | **or n (%)** |  | **or n (%)** |
| **Background characteristics** |  |  |  |  |  |  |
| Participants | 112 | 112 (100.0) | 112 | 112 (100.0) | N/A | N/A |
| Age (years) | 105 | 16.0 (0.4) | 112 | 33.7 (0.4) | N/A | N/A |
| Prior lower limb fracture n (%) | NA | NA | 112 | 20 (17.9) | NA | NA |
| Family history of knee OA n (%) | NA | NA | 112 | 37 (33.0) | NA | NA |
| Ever smoker n (%) | 103 | 60 (53.6) | 112 | 75 (67.0) | 103 | 15 (13.4) |
| Alcohol: Abstinent n (%) | 104 | 64 (57.1) | NA | NA | NA | NA |
| Alcohol: Occasional n (%) | 104 | 34 (30.4) | NA | NA | NA | NA |
| Alcohol: Regular n (%) | 104 | 6 (5.4) | NA | NA | NA | NA |
| Alcohol: g/week | NA | NA | 97 | 31.5 (13.5 - 49.5) | NA | NA |
| Physical activity score | 105 | 16.3 (4.2) | 112 | 14.8 (3.7) | N/A | N/A |
| WOMAC pain (VAS scale) | NA | NA | 111 | 2.1 (4.0) | NA | NA |
| WOMAC stiffness (VAS scale) | NA | NA | 111 | 1.0 (2.0) | NA | NA |
| WOMAC function (VAS scale) | NA | NA | 111 | 4.4 (12.3) | NA | NA |
| WOMAC total (VAS scale) | NA | NA | 111 | 7.3 (17.1) | NA | NA |
| **Clinical characteristics** |  |  |  |  |  |  |
| BMI (kg/m^2^) | 105 | 20.9 (3.5) | 112 | 25.7 (3.6) | 105 | 4.7 (3.0) |
| Height (cm) | 105 | 176.3 (6.4) | 112 | 180.4 (5.8) | 105 | 4.1 (4.2) |
| Weight (kg) | 105 | 65.3 (12.7) | 112 | 83.6 (12.8) | 105 | 18.2 (10.0) |
| Waist circumference (cm) | 105 | 75.2 (8.9) | 112 | 91.8 (10.0) | 105 | 16.5 (8.8) |
| Hip circumference (cm) | 105 | 92.3 (7.3) | 112 | 100.2 (6.4) | 105 | 7.8 (6.4) |
| Systolic blood pressure (mmHg) | 103 | 115.5 (13.0) | 112 | 120.1 (10.4) | 103 | 4.6 (16.2) |
| Diastolic blood pressure (mmHg) | 103 | 69.0 (8.0) | 112 | 76.5 (8.3) | 103 | 7.4 (11.8) |
| Heart rate (bpm) | 103 | 68.5 (9.4) | 112 | 69.9 (13.7) | 103 | 0.9 (16.5) |
| fP-Glucose (mmol/L) | 100 | 5.2 (0.5) | 112 | 5.2 (0.4) | 100 | -0.1 (0.5) |
| fP-Total cholesterol (mmol/L) | 100 | 4.2 (0.9) | 112 | 4.9 (0.9) | 100 | 0.7 (0.7) |
| fP-HDL cholesterol (mmol/L) | 100 | 1.3 (0.3) | 112 | 1.4 (0.3) | 100 | 0.0 (0.2) |
| fP-LDL cholesterol (mmol/L) | 100 | 2.3 (0.6) | 112 | 3.0 (0.8) | 100 | 0.8 (0.6) |
| fp-Triglycerides | 100 | 0.9 (0.6) | 112 | 1.1 (1.0) | 100 | 0.3 (0.9) |
| P-Urate (umol/L) | NA | NA | 112 | 352.5 (58.2) | NA | NA |
| CRP (mg/L) | 102 | 0.5 (0.9) | 112 | 0.5 (0.25 - 1.0) | 102 | 0.4 (1.4) |

**Table S2. Background and clinical characteristics of females of the study population at 16 and 33 years of age and the change in the parameter between the two timepoints.** Data is presented as mean (M) and standard deviation (SD) for normally-distributed continuous variables, median (Mn) and interquartile range (IQR) for skewed continuous variables and count (n) and percentage (%) for count variables. M; Mean, SD; Standard Deviation, OA; osteoarthritis, BMI; Body Mass Index, fP-; fasted plasma, HDL; High-Density Lipoprotein, LDL; Low-Density Lipoprotein, hs-CRP: high-sensitivity C-Reactive Protein.

|  | **At age 16** | | **At age 33** | | **Delta parameter** | |
| --- | --- | --- | --- | --- | --- | --- |
|  | **n** | **M (SD)/Mn(IQR)** | **n** | **M (SD)/Mn(IQR)** | **n** | **M (SD)/Mn(IQR)** |
|  |  | **or n (%)** |  | **or n (%)** |  | **or n (%)** |
| **Background characteristics** |  |  |  |  |  |  |
| Participants | 176 | 176 (100.0) | 176 | 176 (100.0) | N/A | N/A |
| Age (years) | 151 | 16.0 (0.3) | 176 | 33.7 (0.4) | N/A | N/A |
| Prior lower limb fracture n (%) | NA | NA | 175 | 17 (9.7) | NA | NA |
| Family history of knee OA n (%) | NA | NA | 176 | 56 (31.8) | NA | NA |
| Ever smoker n (%) | 159 | 94 (53.4) | 176 | 102 (58.0) | 159 | 8 (4.6) |
| Alcohol: Abstinent n (%) | 161 | 88 (50.0) | NA | NA | NA | NA |
| Alcohol: Occasional n (%) | 161 | 58 (33.0) | NA | NA | NA | NA |
| Alcohol: Regular n (%) | 161 | 15 (8.5) | NA | NA | NA | NA |
| Alcohol: g/week | NA | NA | 157 | 13.5 (7.5 - 31.5) | NA | NA |
| Physical activity score | 160 | 14.8 (3.8) | 176 | 14.8 (3.1) | N/A | N/A |
| WOMAC pain (VAS scale) | NA | NA | 173 | 2.9 (4.3) | NA | NA |
| WOMAC stiffness (VAS scale) | NA | NA | 173 | 1.3 (2.2) | NA | NA |
| WOMAC function (VAS scale) | NA | NA | 173 | 5.4 (9.6) | NA | NA |
| WOMAC total (VAS scale) | NA | NA | 173 | 9.3 (14.9) | NA | NA |
| **Clinical characteristics** |  |  |  |  |  |  |
| BMI (kg/m^2^) | 152 | 21.2 (3.6) | 176 | 25.8 (5.1) | 152 | 4.6 (4.1) |
| Height (cm) | 152 | 164.3 (6.4) | 176 | 165.4 (5.8) | 152 | 1.2 (3.6) |
| Weight (kg) | 155 | 57.2 (10.1) | 176 | 70.7 (15.5) | 155 | 13.8 (10.8) |
| Waist circumference (cm) | 151 | 71.6 (7.8) | 175 | 83.9 (14.0) | 150 | 12.4 (12.2) |
| Hip circumference (cm) | 152 | 93.4 (7.2) | 175 | 99.5 (13.8) | 151 | 6.1 (11.6) |
| Systolic blood pressure (mmHg) | 152 | 117.2 (10.3) | 176 | 106.8 (10.2) | 152 | -10.7 (15.2) |
| Diastolic blood pressure (mmHg) | 152 | 68.8 (6.7) | 176 | 72.8 (9.0) | 152 | 3.8 (11.8) |
| Heart rate (bpm) | 152 | 67.9 (10.5) | 176 | 73.4 (11.1) | 152 | 5.5 (15.4) |
| fP-Glucose (mmol/L) | 136 | 5.1 (1.1) | 175 | 4.9 (0.7) | 135 | 0.2 (0.5) |
| fP-Total cholesterol (mmol/L) | 140 | 4.4 (0.8) | 176 | 4.5 (0.8) | 140 | 0.0 (0.8) |
| fP-HDL cholesterol (mmol/L) | 140 | 1.5 (0.3) | 176 | 1.6 (0.3) | 140 | 0.1 (0.3) |
| fP-LDL cholesterol (mmol/L) | 140 | 2.3 (0.6) | 176 | 2.6 (0.8) | 140 | 0.2 (0.7) |
| fp-Triglycerides | 140 | 0.8 (0.3) | 176 | 0.8 (0.4) | 140 | 0.0 (0.4) |
| P-Urate (umol/L) | NA | NA | 176 | 273.8 (59.4) | NA | NA |
| CRP (mg/L) | 143 | 0.8 (1.6) | 176 | 0.9 (0.5 - 2.0) | 145 | 1.3 (3.8) |

**Table S3. Counts of the most severe in-patient MRI-detected cartilage lesions, BMLs and osteophytes in the tibiofemoral and patellofemoral joint regions.** Data is presented as count (n) and percentage (%)**.** BML; Bone Marrow Lesion.

|  | **Tibiofemoral** | **Tibial medial** | **Tibial lateral** | **Femoral medial** | **Femoral lateral** | **Patellofemoral** | **Any** |
| --- | --- | --- | --- | --- | --- | --- | --- |
| **Cartilage loss** |  |  |  |  |  |  |  |
| 0 (none) | 215 (74.7) | 275 (95.5) | 261 (90.6) | 234 (81.3) | 271 (94.1) | 126 (43.8) | 104 (36.1) |
| 1 (< 10%) | 49 (17.0) | 11 (3.8) | 23 (8.0) | 34 (11.8) | 12 (4.2) | 105 (36.5) | 113 (39.2) |
| 2 (10 - 75%) | 22 (7.6) | 2 (0.7) | 4 (1.4) | 19 (6.6) | 4 (1.4) | 49 (17.0) | 62 (21.5) |
| 3 (> 75%) | 2 (0.7) | 0 (0.0) | 0 (0.0) | 1 (0.3) | 1 (0.3) | 8 (2.8) | 9 (3.1) |
| **Full thickness cartilage loss** |  |  |  |  |  |  |  |
| 0 (none) | 270 (93.8) | 286 (99.3) | 282 (97.9) | 279 (96.9) | 281 (97.6) | 254 (88.2) | 241 (83.7) |
| 1 (< 10%) | 11 (3.8) | 1 (0.3) | 6 (2.1) | 4 (1.4) | 4 (1.4) | 27 (9.4) | 34 (11.8) |
| 2 (10 - 75%) | 6 (2.1) | 1 (0.3) | 0 (0.0) | 4 (1.4) | 3 (1.0) | 6 (2.1) | 11 (3.8) |
| 3 (> 75%) | 1 (0.3) | 0 (0) | 0 (0.0) | 1 (0.3) | 0 (0.0) | 1 (0.3) | 2 (0.7) |
| **Size of BML** |  |  |  |  |  |  |  |
| 0 (none) | 272 (94.4) | 286 (99.3) | 282 (97.9) | 281 (97.6) | 283 (98.3) | 262 (91.0) | 248 (86.1) |
| 1 (< 33%) | 12 (4.2) | 1 (0.3) | 6 (2.1) | 5 (1.7) | 4 (1.4) | 21 (7.3) | 32 (11.1) |
| 2 (33 - 66%) | 3 (1.0) | 1 (0.3) | 0 (0.0) | 1 (0.3) | 1 (0.3) | 4 (1.4) | 6 (2.1) |
| 3 (> 66%) | 1 (0.3) | 0 (0.0) | 0 (0.0) | 1 (0.3) | 0 (0.0) | 1 (0.3) | 2 (0.7) |
| **% of lesion that is BML** |  |  |  |  |  |  |  |
| 0 (none) | 272 (94.4) | 286 (99.3) | 282 (97.9) | 281 (97.6) | 283 (98.3) | 265 (92.0) | 251 (87.2) |
| 1 (< 33%) | 0 (0.0) | 0 (0.0) | 0 (0.0) | 0 (0.0) | 0 (0.0) | 0 (0.0) | 0 (0.0) |
| 2 (33 - 66%) | 1 (1.0) | 0 (0.0) | 0 (0.0) | 1 (0.3) | 0 (0.0) | 1 (0.3) | 1 (0.3) |
| 3 (> 66%) | 15 (5.2) | 2 (0.7) | 6 (2.1) | 6 (2.1) | 5 (1.7) | 22 (7.6) | 36 (12.5) |
| **Osteophytes** |  |  |  |  |  |  |  |
| 0 (none) | 238 (82.6) | 269 (93.4) | 266 (92.4) | 270 (93.8) | 250 (86.8) | 142 (49.3) | 132 (45.8) |
| 1 (small or doubtful) | 41 (14.2) | 14 (4.9) | 17 (5.9) | 13 (4.5) | 32 (11.1) | 127 (44.1) | 135 (46.9) |
| 2 (medium) | 7 (2.4) | 3 (1.0) | 4 (1.4) | 3 (1.0) | 4 (1.4) | 17 (5.9) | 19 (6.6) |
| 3 (large) | 2 (0.7) | 2 (0.7) | 1 (0.3) | 2 (0.7) | 2 (0.7) | 2 (0.7) | 2 (0.7) |

**Table S4. Meniscal morphology, other parameters of interest and prevalence and severity of knee joint effusion in the study population.** Data is presented as count (n) and percentage (%)**.** ACL; Anterior Cruciate Ligament, PCL; Posterior Cruciate Ligament.

|  | **Medial** |  |  | **Lateral** |  | | | |  | |
| --- | --- | --- | --- | --- | --- | --- | --- | --- | --- | --- |
| **Meniscal morphology** | **Anterior** | **Body** | **Posterior** | **Anterior** | **Body** | | | | **Posterior** | |
| Normal | 281 (97.6) | 250 (86.8) | 251 (87.2) | 284 (98.7) | 285 (99.0) | | | | 283 (98.3) | |
| Intrameniscal signal | 3 (1.0) | 21 (7.3) | 22 (7.6) | 1 (0.3) | 0 (0) | | | | 3 (1.0) | |
| Vertical tear | 0 (0) | 0 (0) | 0 (0) | 0 (0) | 0 (0) | | | | 0 (0) | |
| Horizontal tear | 1 (0.3) | 11 (3.8) | 11 (3.3) | 2 (0.7) | 1 (0.3) | | | | 2 (0.7) | |
| Radial tear | 0 (0) | 0 (0) | 0 (0) | 0 (0) | 0 (0) | | | | 0 (0) | |
| Complex tear | 2 (0.7) | 4 (1.4) | 2 (0.7) | 1 (0.3) | 2 (0.7) | | | | 0 (0) | |
| Partial maceration | 1 (0.3) | 2 (0.7) | 2 (0.7) | 0 (0) | 0 (0) | | | | 0 (0) | |
| Total maceration | 0 (0) | 0 (0) | 0 (0) | 0 (0) | 0 (0) | | | | 0 (0) | |
| **Other parameters of interest** | **Present** |  |  |  | |  |  |  | |  |
| ACL tear | 2 (0.7) |  |  |  | |  |  |  | |  |
| ACL repair | 2 (0.7) |  |  |  | |  |  |  | |  |
| PCL tear | 3 (1.0) |  |  |  | |  |  |  | |  |
| PCL repair | 0 (0) |  |  |  | |  |  |  | |  |
| Patellar tendon signal | 12 (4.2) |  |  |  | |  |  |  | |  |
| Any ganglion cyst | 63 (21.9) |  |  |  | |  |  |  | |  |
| Pes anserine bursitis | 2 (0.7) |  |  |  | |  |  |  | |  |
| Infrapatellar bursa signal | 49 (17.0) |  |  |  | |  |  |  | |  |
| Prepatellar bursa signal | 95 (33.0) |  |  |  | |  |  |  | |  |
| Popliteal cyst | 110 (38.2) |  |  |  | |  |  |  | |  |
|  | **None** | **Small** | **Medium** | **Large** | |  |  |  | |  |
| Joint effusion | 165 (57.3) | 99 (34.4) | 21 (7.3) | 3 (1.0) | |  |  |  | |  |

**Table S5.** **Unadjusted Relative Risks or Odds Ratios with 95% Confidence Intervals of selected individual clinical parameters at age 16 for knee MRI findings at age 33.** * Indicates a logistic regression model and Odds Ratio (OR). Unmarked parameters were analyzed with Poisson regression and the result is given as Relative Risk Ratio (RR). CRP; C-reactive protein, LDL; low-density lipoprotein, fP-; fasted plasma, bp; blood pressure.

|  | **hs-CRP (mg/L)** | **LDL chol. (umol/L)** | **fP-glucose(umol/L)** | **Systolic bp (mmHg)** | **Male sex** | **Never smoker** |  |
| --- | --- | --- | --- | --- | --- | --- | --- |
| **Cartilage loss** |  |  |  |  |  |  |  |
| Tibial medial | 1.14 (0.96 - 1.34) | 0.83 (0.25 - 2.73) | 1.01 (0.57 - 1.79) | 0.97 (0.92 - 1.03) | 0.79 (0.26 - 2.40) | 0.43 (0.11 - 1.61) |  |
| Tibial lateral | 0.74 (0.49 - 1.13) | 1.73 (0.83 - 3.58) | 1.09 (0.91 - 1.29) | 0.99 (0.96 - 1.03) | 1.68 (0.79 - 3.57) | 1.05 (0.46 - 2.38) |  |
| Femoral medial | 1.03 (0.96 - 1.21) | 1.36 (0.93 - 2.00) | 0.86 (0.49 - 1.50) | 0.99 (0.97 - 1.01) | 1.11 (0.65 - 1.88) | 0.77 (0.43 - 1.35) |  |
| Femoral lateral | 0.88 (0.65 - 1.21) | 0.74 (0.25 - 2.21) | 0.86 (0.22 - 3.30) | **0.95 (0.91 - 0.99)** | 2.44 (0.89 - 6.75) | 1.17 (0.40 - 3.37) |  |
| Tibiofemoral | 1.02 (0.91 - 1.15) | 1.34 (0.94 - 1.91) | 0.97 (0.75 - 1.25) | 0.98 (0.97 - 1.00) | 1.26 (0.81 - 1.95) | 0.83 (0.52 - 1.33) |  |
| Patellofemoral | 1.04 (0.98 - 1.10) | 1.13 (0.91 - 1.40) | 0.90 (0.74 - 1.11) | 0.99 (0.98 - 1.00) | 0.94 (0.73 - 1.21) | 1.11 (0.85 - 1.45) |  |
| Any | 1.03 (0.98 - 1.09) | 1.10 (0.92 - 1.33) | 0.89 (0.74 - 1.07) | 0.99 (0.98 - 1.00) | 1.04 (0.83 - 1.29) | 0.99 (0.78 - 1.25) |  |
| **FT cartilage loss** |  |  |  |  |  |  |  |
| Tibial lateral* | 0.25 (0.01 - 6.13) | 2.09 (0.58 - 7.55) | 1.08 (0.51 - 2.28) | 0.92 (0.84 - 1.01) | 0.31 (0.06 - 1.72) | 1.41 (0.25 - 7.86) |  |
| Femoral medial | 0.57 (0.19 - 1.66) | 0.60 (0.23 - 1.54) | 1.01 (0.67 - 1.54) | 0.97 (0.91 - 1.02) | 1.05 (0.26 - 4.18) | 0.32 (0.06 - 1.67) |  |
| Femoral lateral | 0.48 (0.10 - 2.35) | 0.62 (0.20 - 1.89) | 1.04 (0.54 - 2.00) | **0.93 (0.87 - 0.98)** | 2.36 (0.49 - 11.28) | 0.48 (0.09 - 2.63) |  |
| Tibiofemoral | 0.60 (0.29 - 1.24) | 1.00 (0.46 - 2.18) | 1.07 (0.85 - 1.36) | **0.95 (0.91 - 0.99)** | 1.57 (0.59 - 4.15) | 0.48 (0.16 - 1.40) |  |
| Patellofemoral | 0.89 (0.75 - 1.05) | 0.84 (0.45 - 1.57) | 0.57 (0.24 - 1.37) | 0.99 (0.96 - 1.02) | 1.18 (0.59 - 2.34) | 1.59 (0.78 - 3.26) |  |
| Any | 0.78 (0.62 - 1.00) | 0.97 (0.60 - 1.58) | 0.75 (0.34 - 1.65) | 0.97 (0.95 - 1.00) | 1.38 (0.78 - 2.45) | 1.13 (0.62 - 2.07) |  |
| **Size of BML** |  |  |  |  |  |  |  |
| Tibial lateral* | 0.50 (0.07 - 3.49) | 2.42 (0.77 - 7.67) | 1.03 (0.44 - 2.41) | 0.96 (0.88 - 1.04) | 0.12 (0.01 - 1.06) | 1.41 (0.25 - 7.86) |  |
| Femoral medial | 0.82 (0.48 - 1.40) | **0.26 (0.10 - 0.67)** | 1.10 (0.89 - 1.36) | 0.99 (0.92 - 1.06) | 1.05 (0.20 - 5.36) | NA |  |
| Femoral lateral | 0.78 (0.34 - 1.79) | 0.73 (0.13 - 3.99) | 1.08 (0.70 - 1.67) | **0.89 (0.84 - 0.94)** | 3.14 (0.51 - 19.36) | 1.43 (0.23 - 8.90) |  |
| Tibiofemoral | 0.77 (0.50 - 1.19) | 1.00 (0.38 - 2.58) | 0.93 (0.53 - 1.66) | 0.96 (0.91 - 1.01) | 1.73 (0.60 - 4.96) | 0.59 (0.18 - 1.97) |  |
| Patellofemoral | 0.81 (0.61 - 1.06) | 0.71 (0.38 - 1.31) | 0.67 (0.28 - 1.62) | 1.00 (0.98 - 1.02) | 1.39 (0.62 - 3.10) | 1.53 (0.66 - 3.53) |  |
| Any | **0.74 (0.56 - 0.99)** | 0.89 (0.53 - 1.48) | 0.71 (0.35 - 1.44) | 0.98 (0.96 - 1.01) | 1.70 (0.91 - 3.20) | 1.14 (0.60 - 2.16) |  |
| **% that is BML** |  |  |  |  |  |  |  |
| Tibial lateral* | 0.50 (0.07 - 3.49) | 2.42 (0.77 - 7.67) | 1.03 (0.44 - 2.41) | 0.96 (0.88 - 1.04) | 0.12 (0.01 - 1.06) | 1.41 (0.25 - 7.86) |  |
| Femoral medial | 0.74 (0.30 - 1.86) | 0.33 (0.10 - 1.02) | 1.10 (0.84 - 1.44) | 0.95 (0.90 - 1.01) | 1.29 (0.29 - 5.68) | NA |  |
| Femoral lateral | 0.88 (0.54 - 1.43) | 0.73 (0.13 - 3.99) | 1.08 (0.70 - 1.67) | **0.88 (0.83 - 0.93)** | 2.36 (0.40 - 13.89) | 0.95 (0.16 - 5.59) |  |
| Tibiofemoral | 0.80 (0.53 - 1.21) | 1.38 (0.56 - 3.41) | 0.98 (0.66 - 1.44) | **0.94 (0.90 - 0.98)** | 2.12 (0.81 - 5.56) | 0.57 (0.18 - 1.77) |  |
| Patellofemoral | 0.82 (0.64 - 1.06) | 0.61 (0.29 - 1.33) | 0.55 (0.20 - 1.49) | 1.00 (0.97 - 1.02) | 1.03 (0.46 - 2.31) | 1.97 (0.86 - 4.53) |  |
| Any | 0.77 (0.59 - 1.00) | 0.93 (0.52 - 1.68) | 0.64 (0.29 - 1.41) | 0.97 (0.95 - 1.00) | 1.52 (0.83 - 2.76) | 1.29 (0.69 - 2.42) |  |
| **Osteophytes** |  |  |  |  |  |  |  |
| Tibial medial | 1.01 (0.79 - 1.30) | 1.29 (0.64 - 2.59) | 0.52 (0.17 - 1.58) | 0.99 (0.97 - 1.02) | 1.15 (0.42 - 3.14) | 1.07 (0.35 - 3.25) |  |
| Tibial lateral | 1.02 (0.85 - 1.22) | 1.00 (0.45 - 2.20) | 0.44 (0.19 - 1.02) | 0.98 (0.95 - 1.01) | 1.18 (0.48 - 2.89) | 0.71 (0.25 - 2.04) |  |
| Femoral medial | 0.99 (0.75 - 1.33) | 1.41 (0.70 - 2.85) | 0.74 (0.20 - 2.68) | 0.97 (0.94 - 1.01) | 1.45 (0.53 - 3.96) | 1.19 (0.40 - 3.50) |  |
| Femoral lateral | 0.90 (0.68 - 1.17) | 0.92 (0.50 - 1.71) | 0.53 (0.25 - 1.12) | 1.00 (0.98 - 1.02) | 1.01 (0.52 - 1.95) | 1.50 (0.75 - 2.99) |  |
| Tibiofemoral | 0.97 (0.81 - 1.17) | 1.10 (0.69 - 1.76) | 0.66 (0.32 - 1.39) | 0.99 (0.97 - 1.01) | 1.17 (0.67 - 2.04) | 1.23 (0.68 - 2.22) |  |
| Patellofemoral | 1.01 (1.93 - 1.08) | 1.14 (0.91 - 1.43) | 0.99 (0.86 - 1.15) | 0.99 (0.98 - 1.00) | **1.41 (1.10 - 1.81)** | 1.23 (0.94 - 1.61) |  |
| Any | 1.03 (1.98 - 1.08) | 1.14 (0.93 - 1.40) | 1.00 (0.89 - 1.12) | 0.99 (0.98 - 1.00) | **1.36 (1.07 - 1.72)** | 1.17 (0.91 - 1.51) |  |
| **Meniscal Tear** |  |  |  |  |  |  |  |
| Med. body horizontal* | 0.76 (0.29 - 1.95) | **3.35 (1.31 - 8.58)** | 0.70 (0.16 - 3.18) | 0.96 (0.90 - 1.02) | 0.76 (0.22 - 2.54) | 0.84 (0.25 - 2.81) |  |
| Med. posterior horizontal* | 1.01 (0.64 - 1.60) | **2.85 (1.10 - 7.34)** | 0.50 (0.12 - 2.09) | 0.99 (0.94 - 1.05) | 0.76 (0.22 - 2.54) | 0.84 (0.25 - 2.81) |  |
| **Other** |  |  |  |  |  |  |  |
| Patellar tend. signal* | 0.50 (0.10 - 2.44) | 1.17 (0.39 - 3.47) | 0.48 (0.12 - 1.85) | 0.97 (0.91 - 1.02) | 1.29 (0.38 - 4.37) | 1.24 (0.35 - 4.34) |  |
| Any ganglion cyst * | 1.10 (0.90 - 1.35) | 1.30 (0.79 - 2.15) | 1.30 (0.87 - 1.94) | 1.01 (0.99 - 1.04) | 1.24 (0.69 - 2.22) | 1.05 (0.58 - 1.90) |  |
| Inf.pat. bursa signal* | 0.84 (0.56 - 1.24) | 1.10 (0.63 - 1.94) | 0.70 (0.33 - 1.48) | 0.99 (0.96 - 1.01) | 0.82 (0.44 - 1.53) | 1.77 (0.87 - 3.57) |  |
| Prepat. bursa signal* | 1.04 (0.86 - 1.26) | 0.89 (0.56 - 1.43) | 0.83 (0.50 - 1.36) | 0.98 (0.96 - 1.00) | 1.30 (0.78 - 2.17) | 1.03 (0.61 - 1.74) |  |
| Joint effusion | 1.01 (1.91 - 1.13) | 1.01 (0.77 - 1.32) | 0.96 (0.79 - 1.16) | 0.98 (0.96 - 0.99) | **1.57 (1.17 - 2.11)** | 1.20 (0.87 - 1.64) |  |
| Popliteal cyst* | 0.90 (0.72 - 1.13) | 1.27 (0.82 - 1.97) | 0.95 (0.68 - 1.31) | 0.99 (0.97 - 1.01) | 0.77 (0.47 - 1.25) | 1.35 (0.81 - 2.24) |  |

**Table S6. Unadjusted Relative Risks or Odds Ratios with 95% Confidence Intervals of the change in selected individual clinical parameters from age 16 to 33 for knee MRI findings at age 33.** Unmarked parameters were analyzed with Poisson regression and the result is given as Relative Risk Ratio (RR). BML; Bone Marrow Lesion, FT; Full thickness, LDL; low-density lipoprotein, cx.; circumference.

|  |  | **ΔWaist cx. (cm)** | **ΔHip cx. (cm)** | **ΔLDL chol. (umol/L)** |
| --- | --- | --- | --- | --- |
| **Cartilage loss** | Tibial medial | **1.063 (1.020 - 1.108)** | **1.071 (1.018 - 1,128)** | 1.352 (0.818 - 2.234) |
|  | Tibial lateral | 1.014 (0.980 - 1.049) | 1.008 (0.963 - 1.055) | 1.239 (0.731 - 2.100) |
|  | Femoral medial | 1.010 (0.985 - 1.036) | 1.010 (0.978 - 1.042) | 1.136 (0.799 - 1.615) |
|  | Femoral lateral | 1.020 (0.975 - 1.066) | 1.029 (0.976 - 1.085) | 1.421 (0.925 - 2.180) |
|  | Tibiofemoral | 1.005 (0.985 - 1.025) | 1.006 (0.982 - 1.030) | 1.014 (0.759 - 1.354) |
|  | Patellofemoral | 0.996 (0.986 - 1.005) | 0.999 (0.988 - 1.010) | 1.020 (0.848 - 1.228) |
|  | Any | 0.996 (0.987 - 1.004) | 1.000 (0.990 - 1.010) | 0.993 (0.850 - 1.161) |
| **FT cartilage loss** | Tibial lateral* | 1.001 (0.924 - 1.085) | 0.983 (0.922 - 1.047) | 1.079 (0.324 - 3.595) |
|  | Femoral medial | 1.029 (0.958 - 1.106) | 1.060 (0.965 - 1.165) | 1.687 (0.668 - 4.263) |
|  | Femoral lateral | 1.044 (1.980 - 1.013) | 1.053 (0.984 - 1.128) | 1.676 (0.920 - 3.052) |
|  | Tibiofemoral | 1.015 (0.973 - 1.059) | 1.034 (0.970 - 1.102) | 1.565 (0.843 - 2.908) |
|  | Patellofemoral | 0.979 (0.954 - 1.004) | 0.990 (0.956 - 1.026) | 0.835 (0.462 - 1.511) |
|  | Any | 0.989 (0.964 - 1.015) | 0.998 (0.964 - 1.033) | 1.021 (0.638 - 1.632) |
| **Size of BML** | Tibial lateral* | 1.010 (0.935 - 1.090) | 0.985 (0.926 - 1.049) | 1.305 (0.447 - 3.815) |
|  | Femoral medial | 1.045 (0.986 - 1.107) | **1.104 (1.005 - 1.214)** | **2.621 (1.182 - 5.815)** |
|  | Femoral lateral | 1.005 (0.975 - 1.036) | 1.014 (0.981 - 1.048) | 0.868 (0.627 - 1.202) |
|  | Tibiofemoral | 1.034 (0.991 - 1.079) | 1.062 (0.990 - 1.139) | **1.933 (1.043 - 3.582)** |
|  | Patellofemoral | 0.983 (0.958 - 1.010) | 0.996 (0.956 - 1.038) | 0.918 (0.512 - 1.648) |
|  | Any | 0.999 (0.971 - 1.028) | 1.012 (0.966 - 1.059) | 1.169 (0.731 - 1.869) |
| **% that is BML** | Tibial lateral* | 1.010 (0.935 - 1.090) | 0.985 (0.926 - 1.049) | 1.305 (0.447 - 3.815) |
|  | Femoral medial | 1.026 (0.939 - 1.121) | 1.044 (0.937 - 1.164) | 1.639 (0.672 - 3.996) |
|  | Femoral lateral | 0.998 (0.975 - 1.021) | 1.010 (0.976 - 1.046) | 0.868 (0.627 - 1.202) |
|  | Tibiofemoral | 1.012 (0.975 - 1.050) | 1.015 (0.968 - 1.064) | 1.389 (0.783 - 2.465) |
|  | Patellofemoral | 0.982 (0.954 - 1.010) | 0.988 (0.958 - 1.019) | 0.687 (0.388 - 1.218) |
|  | Any | 0.992 (0.967 - 1.019) | 0.995 (0.968 - 1.022) | 0.895 (0.583 - 1.373) |
| **Osteophytes** | Tibial medial | 1.032 (0.979 - 1.087) | 1.052 (0.987 - 1.121) | 1.354 (0.819 - 2.240) |
|  | Tibial lateral | 1.003 (0.961 - 1.046) | 1.014 (0.958 - 1.074) | 1.083 (0.601 - 1.953) |
|  | Femoral medial | 1.038 (0.988 - 1.091) | 1.044 (0.980 - 1.112) | **1.835 (1.195 - 2.817)** |
|  | Femoral lateral | 0.996 (0.964 - 1.029) | 1.013 (0.972 - 1.056) | 0.984 (0.625 - 1.549) |
|  | Tibiofemoral | 0.995 (0.970 - 1.021) | 1.006 (0.975 - 1.038) | 0.989 (0.677 - 1.443) |
|  | Patellofemoral | 1.003 (0.990 - 1.016) | 0.998 (0.985 - 1.012) | 1.073 (0.898 - 1.282) |
|  | Any | 1.001 (0.989 - 1.014) | 0.998 (0.985 - 1.011) | 1.065 (0.904 - 1.256) |
| **Meniscal Tear** | Med. body horizontal* | 1.044 (0.983 - 1.108) | 1.014 (0.943 - 1.091) | 1.052 (0.424 - 2.611) |
|  | Med. posterior horizontal* | 0.996 (0.942 - 1.052) | 0.981 (0.937 - 1.026) | 0.650 (0.245 - 1.720) |
| **Other** | Patellar tend. signal* | 1.032 (0.971 - 1.096) | 1.015 (0.944 - 1.092) | 1.245 (0.512 - 3.027) |
|  | Any ganglion cyst * | 0.981 (0.955 - 1.007) | 0.977 (0.949 - 1.007) | 0.763 (0.495 - 1.177) |
|  | Inf.pat. bursa signal* | 0.994 (0.966 - 1.023) | 0.997 (0.965 - 1.031) | 1.304 (0.825 - 2.062) |
|  | Prepat. bursa signal* | 1.005 (0.982 - 1.030) | 1.018 (0.988 - 1.049) | 0.918 (0.629 - 1.341) |
|  | Joint effusion | **1.022 (1.006 - 1.037)** | **1.020 (1.004 - 1.038)** | **1.384 (1.115 - 1.716)** |
|  | Popliteal cyst* | 1.000 (0.978 - 1.023) | 0.992 (0.967 - 1.017) | 1.120 (0.785 - 1.597) |

**Table S7. Clinical data at age 16, 33 and the change in these parameters from age 16 to 33 presented in BMI classes at the age of 16.** fP-; fasted plasma, hs-CRP; high-sensitivity C-Reactive Protein, bp; blood pressure, BMI; Body Mass Index, HDL; High-Density Lipoprotein, LDL; Low-Density Lipoprotein.

|  | At age 16 | | | At age 33 | | | Delta from 16 to 33 | | |
| --- | --- | --- | --- | --- | --- | --- | --- | --- | --- |
| Variable M(SD) | BMI_16_ < 20 | BMI_16_ 20 - 25 | BMI_16_ > 25 | BMI_16_ < 20 | BMI_16_ 20 - 25 | BMI_16_ > 25 | BMI_16_ < 20 | BMI_16_ 20 - 25 | BMI_16_ > 25 |
| Participants n (%) | 105 (42.5) | 116 (46.9) | 26 (10.6) | 105 (42.5) | 116 (46.9) | 26 (10.6) | NA | NA | NA |
| Males n (%) | 50 (43.5) | 43 (37.1) | 12 (46.2) | 50 (43.5) | 43 (37.1) | 12 (46.2) | NA | NA | NA |
| Females n (%) | 65 (56.6) | 73 (62.9) | 14 (53.8) | 65 (56.6) | 73 (62.9) | 14 (53.8) | NA | NA | NA |
| BMI (kg/m^2^) | 18.6 (1.1) | 21.9 (1.4) | 28.8 (4.9) | 23.5 (3.2) | 26.6 (4.1) | 31.7 (4.5) | 4.9 (3.0) | 4.7 (3.7) | 2.9 (5.5) |
| Waist circumference (cm) | 68.1 (3.6) | 73.9 (5.0) | 91.2 (9.9) | 82.4 (12.2) | 88.4 (11.4) | 102.4 (12.2) | 14.4 (11.3) | 14.4 (10.4) | 11.2 (13.1) |
| Hip circumference (cm) | 88.1 (3.5) | 94.6 (4.7) | 106.9 (7.5) | 95.3 (11.3) | 101.7 (9.5) | 111.2 (10.0) | 7.2 (10.9) | 7.0 (8.6) | 4.3 (9.9) |
| Height (cm) | 169.2 (8.3) | 168.8 (8.4) | 171.3 (11.3) | 171.9 (9.6) | 170.7 (9.1) | 174.5 (9.4) | 2.8 (3.4) | 1.9 (3.3) | 3.3 (8.1) |
| Weight (kg) | 53.3 (5.8) | 62.6 (7.7) | 84.0 (13.8) | 69.7 (12.1) | 77.8 (14.2) | 96.7 (16.1) | 16.5 (9.2) | 15.2 (10.8) | 12.7 (15.5) |
| Systolic blood pressure (mmHg) | 116.2 (10.8) | 117.8 (12.1) | 110.7 (9.9) | 111.4 (11.5) | 112.3 (12.4) | 116.1 (14.0) | -4.8 (16.3) | -5.7 (17.9) | 5.4 (17.5) |
| Diastolic blood pressure (mmHg) | 69.4 (7.1) | 68.3 (7.0) | 69.3 (9.1) | 73.9 (7.9) | 74.4 (9.1) | 77.5 (9.6) | 4.4 (11.5) | 5.6 (12.0) | 8.2 (12.6) |
| Heart rate (bpm) | 69.5 (9.3) | 67.5 (11.0) | 67.2 (9.4) | 73.4 (13.3) | 70.3 (10.6) | 72.6 (13.6) | 3.4 (16.8) | 2.5 (14.9) | 5.4 (15.9) |
| fP-Glucose (mmol/L) | 5.1 (0.5) | 5.2 (1.2) | 5.2 (0.5) | 4.9 (0.4) | 5.0 (0.8) | 5.1 (0.5) | 0.5 (0.8) | 0.2 (0.9) | 0.2 (0.8) |
| fP-Total cholesterol (mmol/L) | 4.2 (0.6) | 4.4 (0.9) | 4.6 (1.0) | 4.7 (0.9) | 4.6 (0.9) | 4.8 (1.2) | 0.0 (0.3) | 0.1 (0.3) | 0.1 (0.3) |
| fP-HDL cholesterol (mmol/L) | 2.2 (0.5) | 2.4 (0.6) | 2.6 (0.7) | 1.5 (0.3) | 1.5 (0.3) | 1.4 (0.3) | 0.6 (0.7) | 0.4 (0.8) | 0.2 (0.7) |
| fP-LDL cholesterol (mmol/L) | 1.5 (0.3) | 1.4 (0.3) | 1.3 (0.2) | 2.8 (0.8) | 2.7 (0.8) | 2.9 (0.9) | 0.2 (0.5) | 0.1 (0.4) | 0.4 (1.7) |
| fp-Triglycerides | 0.8 (0.4) | 0.8 (0.3) | 1.1 (0.7) | 0.9 (0.6) | 0.8 (0.4) | 1.4 (1.6) | -0.2 (0.5) | -0.1 (0.6) | -0.1 (0.6) |
| CRP (mg/L) | 0.4 (0.8) | 0.7 (1.6) | 1.3 (1.5) | 1.4 (2.3) | 1.4 (2.8) | 3.1 (5.5) | 1.0 (2.4) | 0.7 (3.1) | 1.7 (4.8) |

**Table S8.** **Clinical data at age 16, 33 and the change in these parameters from age 16 to 33 presented in classes according to the change in fP-glucose from age 16 to 33.** fP-; fasted plasma, hs-CRP; high-sensitivity C-Reactive Protein, bp; blood pressure, BMI; Body Mass Index, HDL; High-Density Lipoprotein, LDL; Low-Density Lipoprotein.

|  | At age 16 | | | At age 33 | | | Delta from 16 to 33 | | |
| --- | --- | --- | --- | --- | --- | --- | --- | --- | --- |
|  | ΔfP-glucose | | | ΔfP-glucose | | | ΔfP-glucose | | |
| Variable M(SD) | < -0.3 | -0.3 to 0.3 | > 0.3 | < -0.3 | -0.3 to 0.3 | > 0.3 | < -0.3 | -0.3 to 0.3 | > 0.3 |
| Participants n (%) | 82 (34.9) | 119 (50.6) | 34 (14.4) | 82 (34.9) | 119 (50.6) | 34 (14.4) | NA | NA | NA |
| Males n (%) | 29 (35.4) | 52 (43.7) | 19 (55.9) | 29 (35.4) | 52 (43.7) | 19 (55.9) | NA | NA | NA |
| Females n (%) | 52 (43.7) | 67 (56.3) | 15 (44.1) | 52 (43.7) | 67 (56.3) | 15 (44.1) | NA | NA | NA |
| BMI (kg/m^2^) | 21.1 (4.3) | 21.0 (3.3) | 21.9 (3.3) | **24.2 (4.3)** | **26.1 (4.2)** | **27.8 (5.2)** | 3.2 (3.2) | 5.1 (3.8) | **5.9 (3.9)** |
| Waist circumference (cm) | 93.1 (7.8) | 92.6 (7.1) | 94.5 (7.3) | 82.7 (15.0) | 88.4 (11.3) | 93.4 (13.0) | **9.9 (12.0)** | **15.8 (10.3)** | **17.6 (10.6)** |
| Hip circumference (cm) | 72.8 (8.8) | 72.6 (8.3) | 75.8 (8.9) | 96.5 (14.6) | 100.9 (9.1) | 103.5 (10.5) | 3.2 (12.3) | 8.3 (8.5) | 8.9 (7.2) |
| Height (cm) | 168.2 (9.4) | 169.5 (8.3) | 171.9 (8.4) | 170.8 (9.7) | 172.0 (9.5) | 173.5 (8.8) | 2.6 (5.2) | 2.5 (2.9) | 1.7 (2.7) |
| Weight (kg) | 59.7 (12.7) | 60.5 (11.8) | **64.8 (11.4)** | 71.2 (16.1) | 77.3 (13.7) | 84.2 (18.0) | **11.5 (9.2)** | **16.8 (10.9)** | **19.4 (11.6)** |
| Systolic blood pressure (mmHg) | 116.0 (11.6) | 116.5 (10.9) | 116.9 (14.3) | 111.7 (14.1) | 112.3 (10.2) | 117.2 (12.3) | -4.0 (20.2) | -4.8 (15.7) | 0.5 (15.8) |
| Diastolic blood pressure (mmHg) | 68.7 (7.1) | 68.8 (6.6) | 70.3 (9.2) | 73.7 (9.6) | 74.4 (7.8) | 77.9 (9.4) | 5.1 (13.2) | 5.0 (10.9) | 7.6 (12.2) |
| Heart rate (bpm) | 66.7 (10.0) | 69.5 (10.8) | 70.0 (9.4) | 72.5 (12.9) | 71.5 (12.5) | 71.0 (11.1) | 5.0 (16.7) | 1.8 (16.0) | 1.1 (15.0) |
| fP-Glucose (mmol/L) | 4.4 (0.8) | 4.3 (0.8) | 4.3 (0.8) | 4.5 (1.0) | 4.7 (0.9) | 4.9 (0.8) | 0.1 (0.8) | 0.4 (0.9) | 0.6 (0.9) |
| fP-Total cholesterol (mmol/L) | 1.5 (0.3) | 1.4 (0.3) | 1.4 (0.2) | 1.5 (0.3) | 1.4 (0.3) | 1.5 (0.3) | 0.1 (0.3) | 0.0 (0.3) | 0.1 (0.3) |
| fP-HDL cholesterol (mmol/L) | 2.3 (0.6) | 2.3 (0.6) | 2.4 (0.5) | 2.6 (0.9) | 2.8 (0.8) | 2.9 (0.7) | 0.3 (0.7) | 0.5 (0.8) | 0.6 (0.7) |
| fP-LDL cholesterol (mmol/L) | 0.8 (0.4) | 0.8 (0.5) | 0.8 (0.5) | 0.8 (0.5) | 0.9 (0.5) | 1.4 (1.5) | 0.0 (0.5) | 0.1 (0.5) | 0.5 (1.3) |
| fp-Triglycerides | 5.4 (1.3) | 5.1 (0.4) | 4.7 (0.5) | 4.8 (0.9) | 5.0 (0.4) | 5.3 (0.4) | -0.6 (0.5) | -0.1 (0.2) | 0.6 (0.5) |
| CRP (mg/L) | 0.7 (1.2) | 0.6 (1.0) | 1.0 (2.4) | 2.0 (4.1) | 1.3 (2.5) | 1.7 (2.3) | 1.3 (3.8) | 0.8 (2.6) | 0.7 (3.0) |
